# Identifying data for the empirical assessment of law (IDEAL): a realist approach to research gaps on the health effects of abortion law

**DOI:** 10.1136/bmjgh-2021-005120

**Published:** 2021-06-11

**Authors:** Scott Burris, Adrienne R Ghorashi, Lindsay Foster Cloud, Rachel Rebouché, Patty Skuster, Antonella Lavelanet

**Affiliations:** 1 Center for Public Health Law Research, Beasley School of Law, Temple University, Philadelphia, Pennsylvania, USA; 2 Ipas, Chapel Hill, North Carolina, USA; 3 Department of Sexual and Reproductive Health and Research and UNDP-UNFPA-UNICEF-WHO-World Bank Special Programme of Research, Development and Research Training in Human Reproduction (HRP), World Health Organization, Geneve, GE, Switzerland

**Keywords:** health policies and all other topics, health policy, maternal health

## Abstract

Reproductive rights have been the focus of United Nations consensus documents, a priority for agencies like the WHO, and the subject of judgments issued by national and international courts. Human rights approaches have galvanised abortion law reform across numerous countries, but human rights analysis is not designed to empirically assess how legal provisions regulating abortion shape the actual delivery of abortion services and outcomes. Reliable empirical measurement of the health and social effects of abortion regulation is vital input for policymakers and public health guidance for abortion policy and practice, but research focused explicitly on assessing the health effects of abortion law and policy is limited at the global level. This paper describes a method for Identifying Data for the Empirical Assessment of Law (IDEAL), to assess potential health effects of abortion regulations. The approach was applied to six critical legal interventions: mandatory waiting periods, third-party authorisation, gestational limits, criminalisation, provider restrictions and conscientious objection. The IDEAL process allowed researchers to link legal interventions and processes that have not been investigated fully in empirical research to processes and outcomes that have been more thoroughly studied. To the extent these links are both transparent and plausible, using IDEAL to make them explicit allows both researchers and policy stakeholders to make better informed assessments and guidance related to abortion law. The IDEAL method also identifies gaps in scientific research. Given the importance of law to public health generally, the utility of IDEAL is not limited to abortion law.

**Summary box**

- Law is an important influence on health, including via the accessibility of abortion services, but too often the health effects of laws and legal practices are not rigorously evaluated.
- Reliable empirical measurement of the effects of abortion regulation is vital input for policymakers and public health guidance for abortion policy and practice, but research assessing the health effects of abortion law and policy is limited at the global level.
- This paper reports on the use of a new method -- Identifying Data for the Empirical Assessment of Law (IDEAL) – that deploys causal modeling to link abortion laws that have not been adequately evaluated in empirical research to abortion processes and outcomes that have been more thoroughly studied.
- IDEAL can help both researchers and policy stakeholders to make better-informed assessments and produce stronger guidance related to abortion and other important areas of law, while also identifying gaps in scientific research.

## Background

Since the mid-1990s, reproductive rights have been the focus of United Nations consensus documents, a priority for the WHO, and the subject of judgments of national and international courts. With 25 million unsafe abortions each year,^1^ an increasing number of international bodies have supported legalisation of abortion and the elimination of legal impediments as essential to the protection of women’s rights to equality, non-discrimination, liberty, health, autonomy and freedom from violence.^2^ International human rights bodies have explicitly called on States to ensure that abortion services are available, accessible and of good quality.^3^ Human rights law also requires that abortion laws are evidence-based and proportionate; thus, states must assess how legal provisions regulating abortion affect abortion services and outcomes.

Reliable empirical measurement of the health and social effects of abortion regulation is vital input for policy-makers and essential for developing public health guidance for abortion policy and practice.^4^ WHO’s evidence-based guideline development process uses an INTEGRATE framework to assess the impacts of all kinds of health interventions.^5^ The balance of health benefits and harms, human rights and sociocultural acceptability, health equity, equality and non-discrimination, societal implications, financial and economic factors, and feasibility and health system elements are all considered. Empirical research focused on the health effects of abortion law and policy is limited at the global level. More such research is urgently needed, but, in the meantime, existing research on better-studied aspects of abortion can shed empirical light on the effects of abortion laws and provide important practical insights for policy.

This paper describes a method developed by the authors, Identifying Data for the Empirical Assessment of Law (IDEAL), to locate evidence on health effects of abortion regulations in existing research that does not explicitly focus on law. Consistent with the WHO definition of health, ‘health effects’ in this project encompassed the full range of physical, mental and socioeconomic outcomes relevant to well-being. In the service of a ‘realist’ policy evaluation approach,^6 7^ the IDEAL method posits a ‘programme theory’ for each law, in the form of a causal logic model setting out events and outcomes that may plausibly occur *assuming* key facts that can and should be investigated in future research: that the law is uniformly enforced, as written, within and across different jurisdictions, and that the healthcare providers and individuals whose conduct is regulated by the law know about and understand the rules. In general, popular knowledge of the precise requirements of law is imperfect, and law as implemented can be very different than law on the books, so the models are stating a theory about causal processes that would occur under specified conditions, not offering generalisable findings about how law actually operates in any particular jurisdiction. Their value lies in identifying evidence that can be useful in making tentative inferences about legal effects in the absence of direct evidence, and in pointing to important research questions. In the absence of direct evidence, the IDEAL process can also serve a precautionary role, by identifying non-trivial legal health risks that legislators should consider when enacting or amending abortion laws.

This work was commissioned as part of the WHO update to the *Safe Abortion: Technical and Policy Guidance for Health Systems*.^8^ The approach was applied to six legal interventions contained in the WHO’s Global Abortion Policy Database,^9^ and identified as critical for review by participants in a technical consultation held by WHO in preparation for the update to the guidelines: mandatory waiting periods, third-party authorisation (including parental involvement, spousal consent and additional approval in cases of sexual assault), gestational limits, criminalisation, provider restrictions and ‘conscientious objection’ (also known as ‘conscientious refusal’). Currently, WHO guidelines make no recommendations related to these legal interventions, but describe them as regulatory and policy barriers that may influence access to timely, safe abortion care.^8^

## Development of the ideal process

Research assessing the health effects of legal interventions has often been important in guiding public health policy, but remains relatively rare for many topics, including reproductive health.^10–12^ Abortion laws, like other legal interventions, operate in a complex and context-dependent manner, with multiple components that may be non-linear in their effects.^13^ Most research studies assessing the effects of law on abortion-related outcomes investigate small populations in single jurisdictions, differ in their definitions of key variables, are subject to design limitations, and focus on the USA.^11 14–16^ IDEAL was intended to support the development of evidence-based guidelines and practices by identifying social science and epidemiological evidence related to abortion that does not explicitly address law, but can nonetheless enhance the understanding of legal effects and identify priority research topics. The challenge posed for the WHO guideline development process was to identify such evidence and provide a transparent, credible explanation for its relevance to an assessment of legal effects.

The research team of academics, lawyers, reproductive health experts and law students developed a three-step process. Step 1 identified empirical research that was designed to assess health effects of abortion laws. The team conducted a rapid scan to retrieve such research on the six types of law included in this project. Search terms for parental involvement laws included *minor, abortion, parental consent, judicial bypass* and *law*. A legal researcher and student researchers independently performed searches in the PubMed database. Each PubMed search was supplemented by a Google search for grey literature. References returned in the search results were reviewed for additional relevant studies. For parental consent, researchers identified 20 individual studies and reviews that explicitly evaluated effects of parental involvement laws on abortion processes or outcomes.

Step 2 developed causal logic models for the six types of legal interventions on abortion to display plausible pathways from the implementation of the restriction to health and socioeconomic outcomes.^6 17^ The research team drew on the studies retrieved in step 1 to design the causal models based on sociolegal theory and processes and effects of law identified in that research. Four ‘common pathways’ appeared repeatedly within these causal models: delayed abortion, increased costs, unintended childbirth and legally prohibited abortion. These common pathways were modelled separately to capture greater detail.

Step 3 used the models as a guide to conduct a second rapid scan. This step aimed to identify non-legal studies investigating whether the processes and outcomes posited in the models do, in fact, occur, and with what frequency, severity or consequence. This evidence, in turn, would support plausible inferences of causality for practical policy and guideline development purposes.^7 18^

## Practical insights: evidence of plausible legal effects

When few studies directly link laws to health or other outcomes, causal modelling is an expeditious way to identify data that measures the effects of processes that law requires or will influence, if implemented as written. We were able to retrieve sufficient evidence to support the development of models for each of the legal interventions included in the study and identify research that illuminated processes (like delay in abortion services) and outcomes (like increasing risk of complications with gestational age) that could result from laws’ application. Table 1 reports selected results for the six legal interventions. For each type of law, table 1 lists the main causal pathways and outcomes we hypothesised based on our research, and provides examples of non-legal research illuminating the pathways we identified. The studies referenced in table 1 were selected as representative of the IDEAL results on the particular abortion restriction, but the list is not exhaustive and reflects limitations of the scan we conducted and the relevant literature generally. Causal models for all the included laws, and additional studies identified by the IDEAL process, appear in the online supplement to this article (online supplemental file). To demonstrate the application of the IDEAL method, we present here detailed findings on parental involvement laws.

10.1136/bmjgh-2021-005120.supp1 Supplementary data

**Table 1:** Causal pathways linking legal regulations of abortion to plausibly related outcomes and relevant research

Legal intervention	Select causal pathways	Plausibly related outcomes	Examples of relevant research identified
Law requires parental involvement or notification for abortion	Minor complies with law and involves parent Minor does not involve parent Minor invokes legal exception or judicial bypass	Intrafamilial conflict Continued pregnancy Lawful abortion Legally prohibited abortion Unintended Childbirth Delayed abortion Increased costs	Henshaw SK, Kost K. Parental involvement in minors' abortion decisions. *Fam Plann Perspect* 1992 Sep-Oct;24(5):196–207, 213. Hasselbacher LA, Dekleva A, Tristan S, Gilliam ML. Factors influencing parental involvement among minors seeking an abortion: a qualitative study. *Am J Public Health*. 2014;104(11):2207–2211. Coleman-Minahan K, Stevenson AJ, Obront E, Hays S. Adolescents obtaining abortion without parental consent: Their reasons and experiences of social support. *Perspect Sex Reprod Health*. 2020;52(1):15–22.
Law requires spousal notification/consent for abortion	Individual notifies spouse and obtains consent Non-compliance with spousal consent Individual invokes legal exception	Lawful abortion Continues pregnancy Unintended childbirth Legally prohibited abortion IPV or marital disharmony Delayed abortion Increased costs	Altshuler, Nguyen *et al*., *Male Partners' Involvement in Abortion Care: A Mixed-Methods Systematic Review*, *Perspect Sex Reprod Health*. 2016 Dec; 48:209–219. Hall M, Chappell LC, Parnell BL, Seed PT, & Bewley S. Associations between intimate partner violence and termination of pregnancy: a systematic review and meta-analysis. PLoS medicine 2014;11(1):e1001581.
Additional authorisation (AA) required for abortion in cases of sexual assault	Pregnant individual complies with AA law and obtains authorisation Pregnant individual is denied authorisation Non-compliance with AA law	Lawful abortion Secondary rape victimisation Legally prohibited abortion Unintended childbirth Delayed abortion Increased costs	Maier SL.“I have heard horrible stories …": rape victim advocates' perceptions of the revictimization of rape victims by the police and medical system. *Violence against women* 2008; 14(7):786–808. Blake M, Drezett J, *et al*. Factors associated with the delay in seeking legal abortion for pregnancy resulting from rape. *International Archives of Medicine* 2015;8. doi:10.3823/1628.
Law requires a mandatory waiting period between clinical encounter and abortion	Non-compliance with waiting period Individual eligible for legal exception to waiting period Compliance with waiting period	Unintended childbirth Legally prohibited abortion Lawful abortion Delayed abortion Increased costs	Karasek, D., Roberts, S. C., & Weitz, T. A. (2016). *Abortion Patients Experience and Perceptions of Waiting Periods: Survey Evidence before Arizona’s Two-visit 24-hour Mandatory Waiting Period Law*. Womens Health Issues, 26(1), 60–66. doi:10.1016/j.whi.2015.10.004 Bartlett, L.A., *et al*., Risk factors for legal induced abortion-related mortality in the United States. *Obstet Gynecol*, 2004. 103(4): p. 729–37.
Law sets gestational age limits for obtaining abortion	Pregnancy deemed to exceed legal limits Pregnancy deemed within legal limits Individual eligible for legal exception to gestational limit	Legally prohibited abortion Unintended childbirth Lawful abortion Delayed abortion Increased costs	Upadhyay, U.D., Weitz, T.A., Jones, R.K., Barar, R.E., & Greene Foster, D. *Denial of Abortion Because of Provider Gestational Age Limits in the United States*, 104 Am J Public Health,1687–1694 (2014) Henshaw, S.K. & Finer, L. The Accessibility of Abortion Services in the United States, 2001. *Perspectives on Sexual and Reproductive Health*, Guttmacher Institute, 2003 Jan. –Feb.; 35(1): 16–24.
Law limits the types of healthcare professionals authorised to perform abortions	Availability and accessibility of abortion services Changes in health workforce training and services infrastructure	Lawful abortion Legally prohibited abortion Delayed abortion Increased costs Unintended childbirth	Grimes DA. *Clinicians who provide abortions: the thinning ranks*. Obstet Gynecol. 1992 Oct;80(4):719–23. PMID: 1 407 901. Joffe C, Yanow S. *Advanced practice clinicians as abortion providers: current developments in the United States*. Reprod Health Matters. 2004;12(Suppl):198–206. doi: 10.1016/S0968-8080(04)24008-.
Law criminalises some or all abortions	Availability and accessibility of abortion services Changes in health workforce training and services infrastructure Arrest and prosecution of individuals obtaining abortion outside formal health system	Lawful abortion Legally prohibited abortion Delayed abortion Increased costs Unintended childbirth Increased abortion stigma Harm to the confidentiality of patient-provider relationships	Baum, S., DePiñeres, T., & Grossman, D. (2015). Delays and barriers to care in Colombia among women obtaining legal first- and second-trimester abortion. *International journal of gynaecology and obstetrics*, 131(3), 285–288. Hanschmidt, F., Linde, K., Hilbert, A., Riedel-Heller, S. G., & Kersting, A. (2016). Abortion Stigma: A Systematic Review. *Perspectives on sexual and reproductive health*, 48(4), 169–177.
Law allows medical provider or facility to refuse to perform an abortion due to conscientious objection	Availability and accessibility of abortion services Changes in health workforce training and services infrastructure Provider referral behaviour	Lawful abortion Legally prohibited abortion Unintended childbirth Delayed abortion Increased costs	Awoonor-Williams, J. K., Baffoe, P., Aboba, M., Ayivor, P., Nartey, H., Felker, B., Van der Tak, D., & Biney, A. (2020). Exploring Conscientious Objection to Abortion Among Health Providers in Ghana. *International perspectives on sexual and reproductive health*, 46, 51–59. Turner, K. L., Pearson, E., George, A., & Andersen, K. L. (2018). Values clarification workshops to improve abortion knowledge, attitudes and intentions: a pre-post assessment in 12 countries. *Reproductive health*, 15(1), 40.

### The parental involvement model

**Figure 1:**
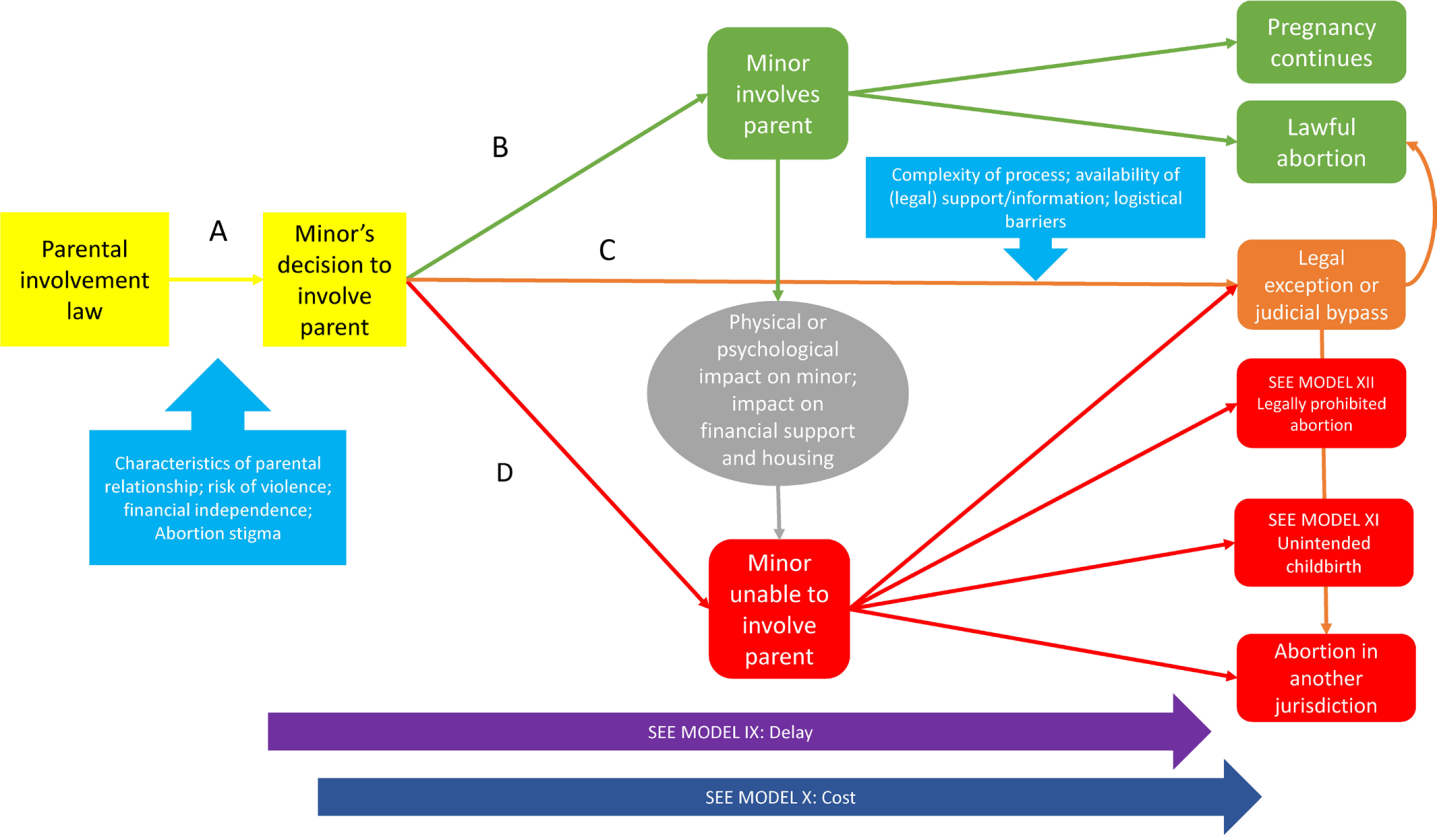
Parental involvement law.

Parental involvement laws in 51 countries require a minor to notify one or both parents and/or obtain their consent before they can lawfully obtain an abortion.^9^ These laws typically also provide for an alternative approval process involving judges or other persons, which we will refer to as a ‘bypass’. Studies directly addressing the impact of parental involvement laws, primarily in US settings,^16^ pointed to several generic causal pathways from the implementation of mandatory parental involvement for minors’ abortion to health outcomes. See figure 1.

Pathway A depicts the options for the pregnant minor: parental involvement as required, a bypass if permitted, or non-compliance. The choice may be influenced by such factors as the relationship with the parents, the practical need for assistance, or fear of the parental reaction.^19^ Pathway B depicts a minor notifying a parent, which can clear the minor’s path to obtaining an abortion or lead to a decision to proceed with the pregnancy. The model also depicts the impact of parental involvement on the health and socioeconomic well-being of the minor, drawing on evidence of parental involvement’s possible positive effects^19 20^ and its potential to produce intrafamilial conflict and other negative consequences for the minor.^21 22^ Such conflict may lead to the minor experiencing an undesired pregnancy that proceeds to childbirth or a legally prohibited abortion, or seeking judicial authorisation where available.

Pathway C represents a minor’s decision to pursue a legal alternative to parental consent or notification, such as seeking judicial approval of an abortion. Accessibility of this option is mediated by the complexity of the alternative process and availability of legal or other assistance services.^23 24^ Some minors may be unable to complete the process, shifting to the parental involvement or non-compliance pathways.^25–27^ Should the alternative procedure not lead to a lawful abortion, the minor may give birth, obtain a legally prohibited abortion or shift to the parental involvement pathway. Pathway D represents the minor’s non-compliance with the parental involvement law, leading to an unintended birth, legally prohibited abortion or an abortion in another jurisdiction.

The causal model shown in figure 1 was derived from primarily qualitative and survey-based studies that explored how parental involvement influenced minors’ abortion choices and trajectories. Studies of health outcomes directly testing effects of law were almost entirely missing, but the model in figure 1 makes the connection between observed behaviour related to the law and a set of common pathways with known health and social consequences. These, as shown in figure 1, include obtaining a legally prohibited abortion, unintended childbirth, delay in obtaining an abortion, and increased cost.

### The delayed abortion model

**Figure 2:**
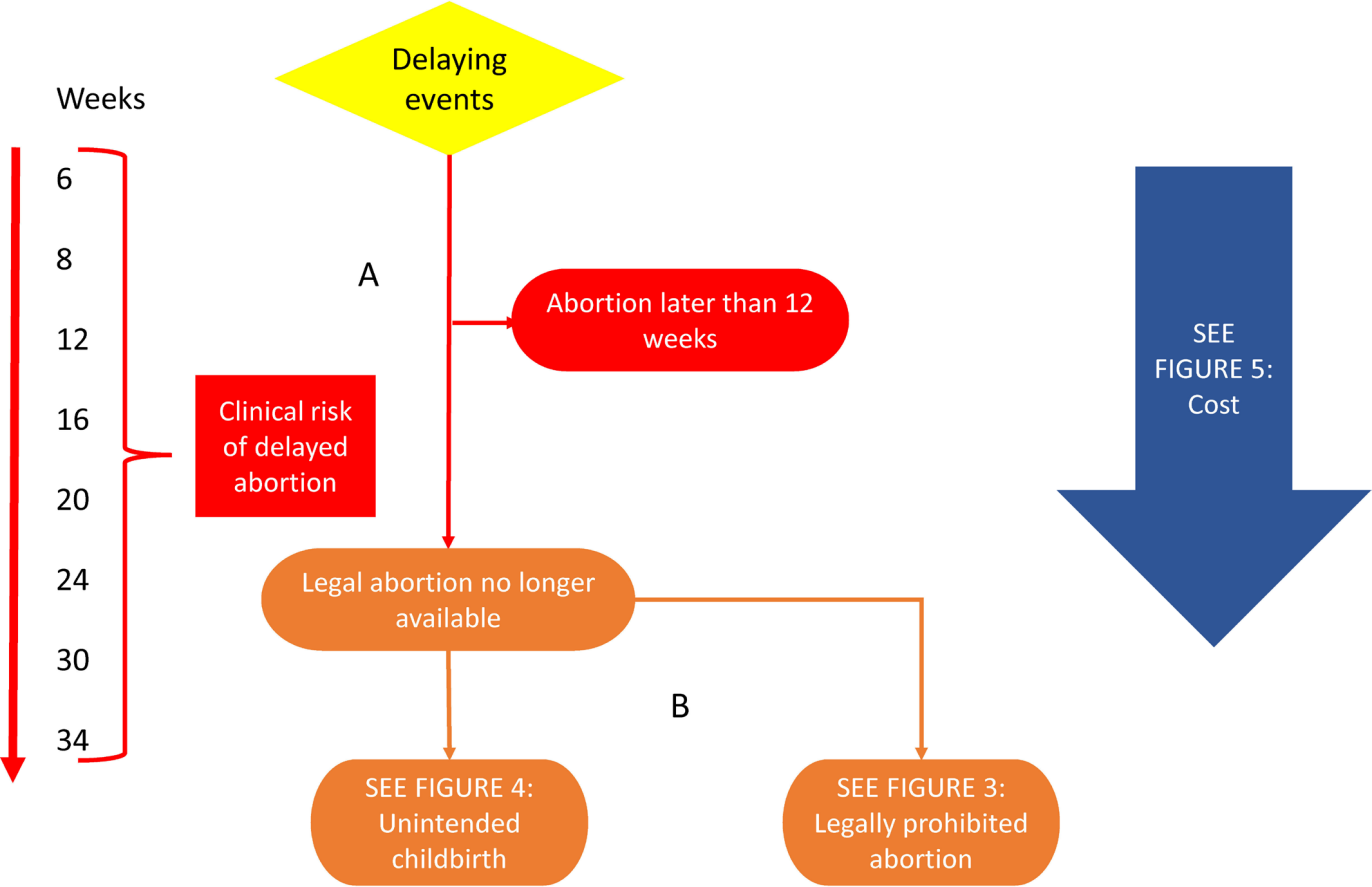
Delayed abortion.

Figure 2 expands the model in figure 1 to link parental involvement law to evidence of the effects of delayed abortion. Parental involvement laws are associated with delay in receiving abortion services.^16^ Pathway A connects legal delay to epidemiological evidence of the rising risk of maternal mortality as gestational age increases^28^; although the absolute risk is quite low, the increase in relative risk has been reported to be as high as 38% for each additional week of gestation.^29^ By causing the use of more expensive surgical or medical procedures at later gestations, or the unintended birth of a child, delay can also increase costs (pathways B). Travel to a different location where law provides access to abortion is a well-identified way to overcome legal barriers of all kinds, and can also occasion delay and additional cost.^30^

### The unintended childbirth model

**Figure 3:**
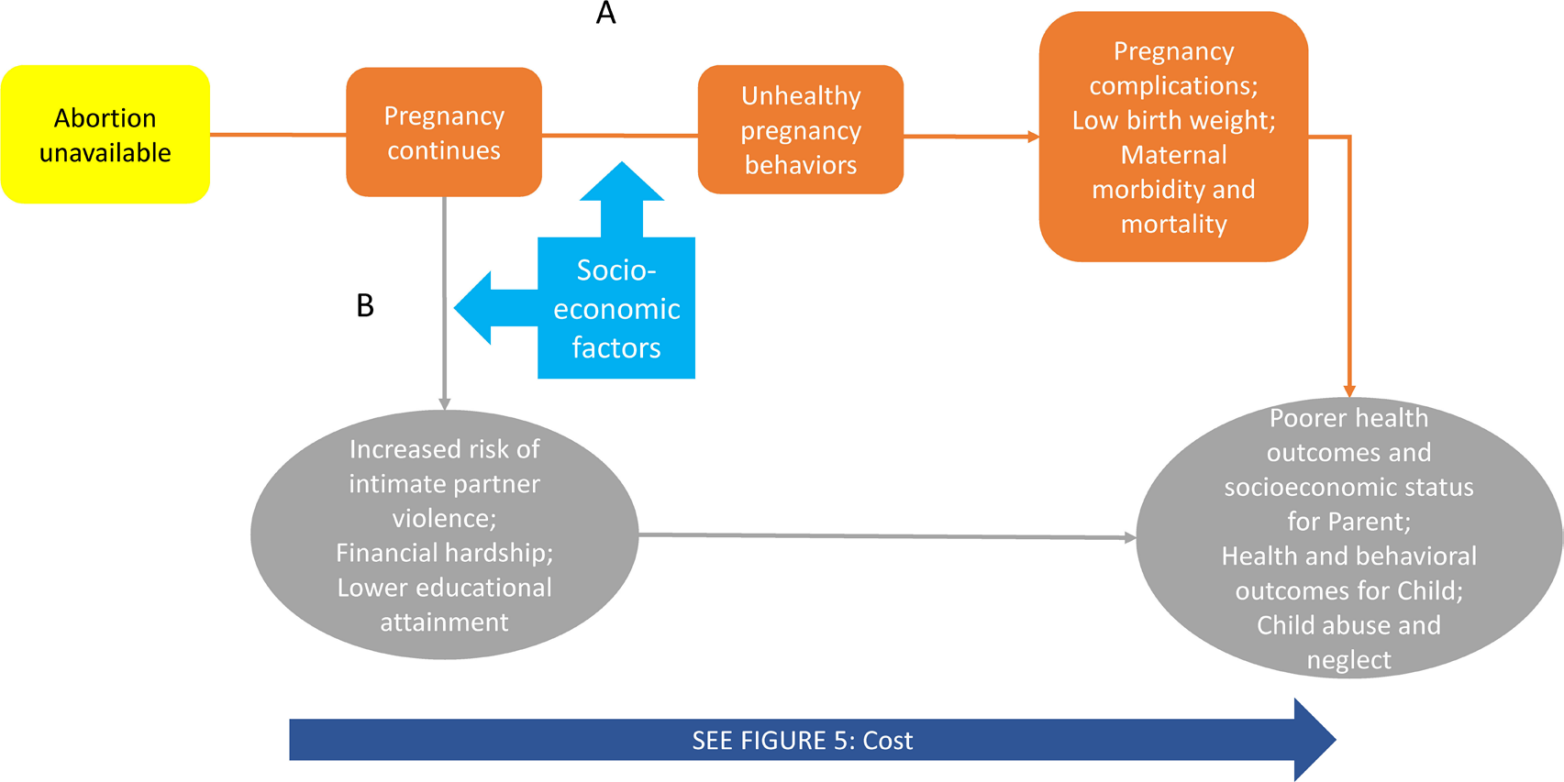
Unintended childbirth.

Figure 3 connects parental involvement laws to well-identified negative health and socioeconomic outcomes of unintended childbirth. Pathway A shows law’s logical connection to the known risks of poorer health outcomes in adolescents carrying an unintended pregnancy to term.^31–34^ Poorer maternal health outcomes may arise from socially mediated unhealthy pregnancy behaviour and lack of access to prenatal care for adolescents.^32^ Additional documented negative health effects for pregnant individuals and their families may include lower socioeconomic status and increased risk of abuse (pathway B). Even a healthy pregnancy and birth may entail increased risk of intimate partner violence, financial distress and lower educational attainment.^35–37^ Both pathways reflect the increased costs associated with carrying an unwanted pregnancy to term.

### The Legally Prohibited Abortion Model

**Figure 4:**
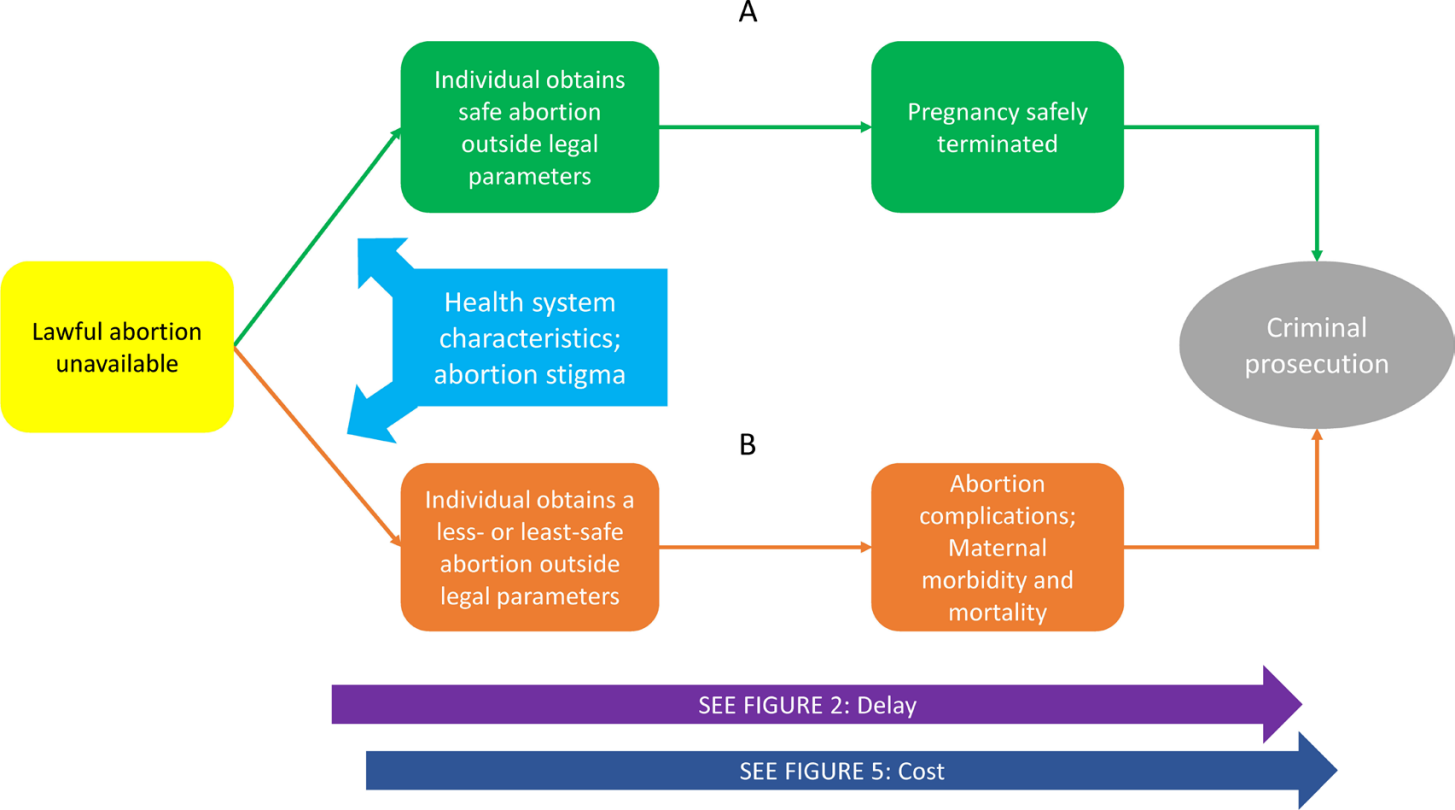
Legally prohibited abortion.

Figure 4 connects parental involvement laws to the processes and outcomes related to a legally prohibited abortion. Pathway A describes a pregnant individual who does not qualify for a legal abortion but is able to obtain a safe abortion outside of legal requirements. A self-managed abortion by a person who has the necessary information, properly using the combination of mifepristone and misoprostol, is considered to be a safe abortion. The social and abortion service-delivery environment, including the availability of willing providers,^38 39^ availability of quality medicines,^40^ and patient socioeconomic status (SES) may influence whether abortion may be safely obtained outside the law.^41^

Pathway B depicts a pregnant individual’s resort to a less-safe or least-safe abortion.^1^ Abortion stigma is a mediating factor and may influence an individual’s decision to obtain abortion outside of legal requirements and, along with legal penalties, deter them from seeking appropriate care for complications.^42–46^ In both pathways, the individual may be faced with prosecution for violating abortion law, delayed care and increased costs.

### The increased costs model

**Figure 5:**
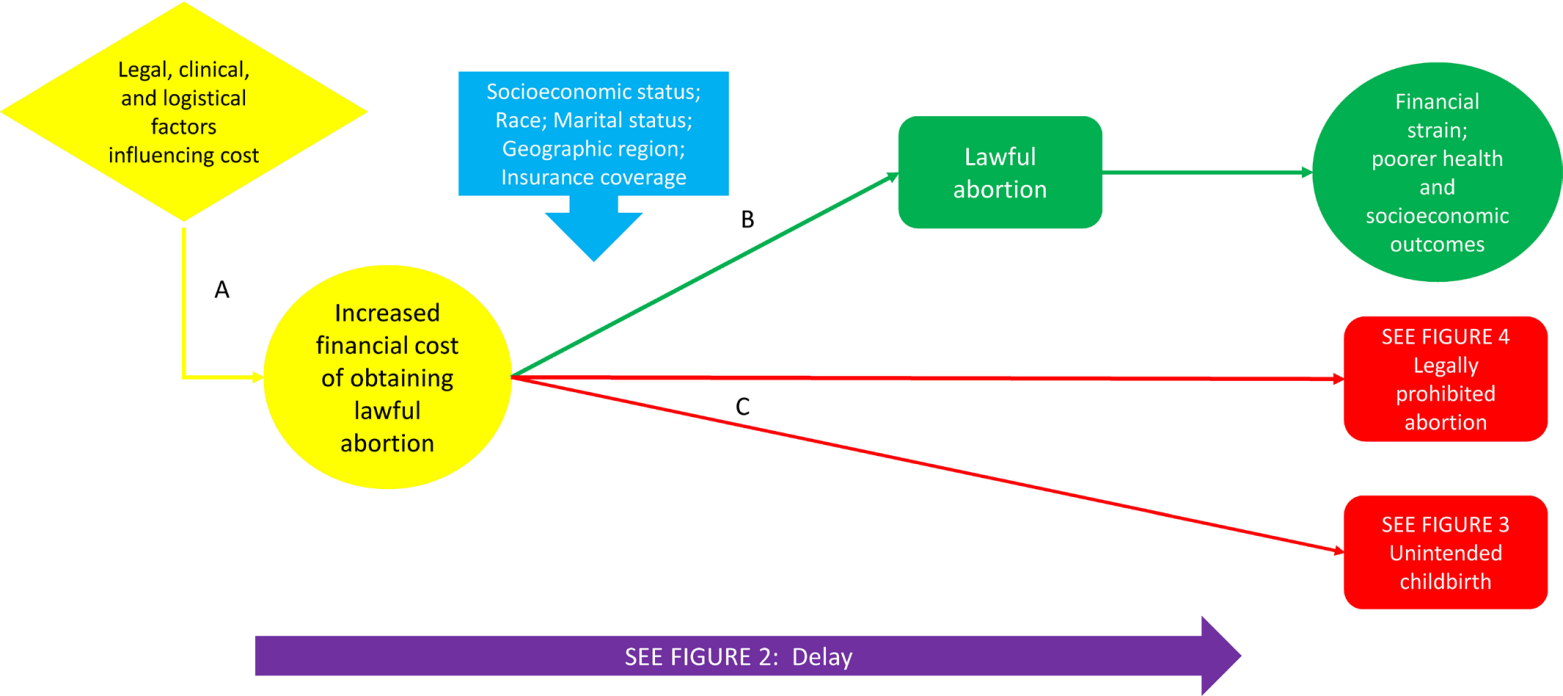
Increased cost.

Cost of an abortion can be a significant barrier to obtaining care and can exacerbate negative health and socioeconomic outcomes for the pregnant individual and their family. In figure 5, pathway A links the impact of legal, clinical and logistical factors depicted in other models on the costs associated with obtaining abortion. The impact of cost is mediated by demographic factors such as SES, marital status and geographical location, as well as insurance coverage.^47^ As shown in pathway B, increased financial cost may not preclude obtaining a lawful abortion, but may entail financial and related stress for the individual. Pathway C depicts inability to obtain an abortion because of cost leading to unintended childbirth or an abortion outside legal parameters. Unintended pregnancy and childbirth can lead to more costs linked to providing necessities for raising a child as well as costs associated with carrying the pregnancy to term, including complications during childbirth such as low birth weight, premature birth, and/or maternal morbidity and mortality. Surmounting the barriers imposed by higher costs may cause delay in obtaining an abortion.

## Discussion

Although parental involvement and other abortion laws can and should be assessed based on their conformity with human rights norms, such an analysis does not in itself provide empirical data on the actual effects of laws and the manner of their implementation. Our study demonstrates that existing studies of good quality can potentially support evidence-based guidance for policy. Strong evidence of the negative health effects of delayed access to care and adolescent health risks points to the importance of expediting or removing legal procedures for parental involvement in minors’ abortion. Recognising that adding law-related costs to abortion care can have disparate health impact highlights the potential link between abortion laws and health inequities. The relevance of well-known risks of legally prohibited abortion are relevant to understanding the possible links between law, delayed care, intrafamilial conflict, and a minor’s inability or unwillingness to pursue legal options. Evidence that undesired childbirth is harmful to both parent and child points to gaps in research on whether parental involvement laws compromise minor’s preferences for parenthood. Thus, results of the IDEAL study have also informed the WHO guideline-development process by populating a research agenda on legal effects and in areas for which legal effects are unclear.

Considering the potential impact of abortion laws on health, studies designed to rigorously evaluate the implementation and effects of abortion restrictions across the globe are too limited. In most countries, there has been no evaluation of these laws’ negative, positive or neutral health implications. Even in the USA, the evidence base often does not parse out health outcomes or disparities associated with legal barriers for specific populations.^48^ However, existing high-quality studies demonstrate that rigorous research on legal effects is possible.^49 50^

The IDEAL method attempts to create an objective framework for crystallising the various influences and consequences attributable to the impact of specific abortion restrictions, leading to the identification of untapped scientific evidence on plausible effects of the law. The framework itself can be applied to a specific law of a country or a subnational jurisdiction, and across topics and fields, where the evaluation of laws and policies is lacking or could otherwise benefit from a more expansive outlook. The IDEAL method could also be used to explore the interaction of multiple types of legal restrictions within a policy environment. Disentangling both the individual mechanisms of a law and the interaction of multiple restrictions can provide a more accurate understanding of how implementation of these laws could be affecting the service delivery environment and related health outcomes and disparities, both positively and negatively. Mapping the cumulative consequences of delay and cost, for example, could illuminate how social position is transformed by apparently non-discriminatory legal interventions into inequitable health outcomes, contributing to the literature theorising and applying the social determinants of health. The utility of IDEAL in these applications is not limited to the realm of abortion law.

As a norm setting agency, WHO has a role in the ‘dissemination of valuable knowledge’.^51^ Considerable knowledge about legal effects is available in research that documents medical and social processes in abortion. By enhancing our understanding of these causal relations and fortifying the evidence base with empirical studies or pointing to gaps in the literature, we pave the way for more informed and targeted policy research. Policy-makers and advocates generally can then use this actionable data to craft evidence-based solutions with a specific lens on improving health outcomes.

## Conclusions

Causal modelling exposes the complex interplay among known variables and outcomes, legal requirements and procedures, and individual and population health. Like other modes of ‘realist’ review, the IDEAL process depends on an existing framework of research related to the phenomena regulated by law, and on transparent logical reasoning backed by established theory. While models can support only guarded causal inferences about actual policy effects in any given legal setting, these causal hypotheses gain evidentiary weight as additional evidence is identified, documenting the occurrence or character of predicted causal pathways. The study also offers a method for illuminating—and to some degree filling—gaps in the evidence base on the impact of abortion laws on significant health, behavioural, and socioeconomic outcomes. The IDEAL method provides plausible and actionable insights that can better inform guidance documents, as well as targeted strategies for research, policy and advocacy.

## Data Availability

All data relevant to the study are included in the article or uploaded as supplementary information.
