# Supplementary material for: Identifying data for the empirical assessment of law (IDEAL): a realist approach to research gaps on the health effects of abortion law
Source: BMJ Glob Health. 2021 Jun 11;6(6):e005120. doi: 10.1136/bmjgh-2021-005120 (PMC8202112; doi:10.1136/bmjgh-2021-005120)
Supplement: Supplementary data [file bmjgh-2021-005120supp001.pdf]

Identifying data for the empirical assessment of law (IDEAL): A realist approach to research gaps on the health effects of abortion law

Data Supplement

TABLE OF CONTENTS

INTRODUCTION **2**

CAUSAL MODELS OF ABORTION LAWS **3**

I. *Parental Involvement in Minor's Abortion* 3
II. *Spousal Consent for Abortion* 6
III. *Additional Authorization* 8
IV. *Gestational Limits* 12
V. *Mandatory Waiting Period Requirement* 14
VI. *Provider Restrictions* 17
VII. *Criminalization of Abortion* 20
VIII. *Conscientious Objection* 22

CAUSAL MODELS OF COMMON PATHWAYS

IX. *Delay* 26
X. *Cost* 28
XI. *Unintended Childbirth* 31
XII. *Legally Prohibited Abortion* 34

Introduction to the Data Supplement

This supplemental document provides detailed results from our study using the IDEAL process (Identifying Data for the Empirical Assessment of Law) investigating six critical legal interventions for abortion: mandatory waiting periods, third-party authorization,[1] gestational limits, criminalization, provider restrictions, and conscientious objection. This supplement contains the full set of causal models developed during the study, along with tables that summarize the causal pathways and provide examples of relevant non-legal studies identified through the research process. The purpose and methods of the IDEAL process and a sample of the results are further described in the published paper.

The IDEAL study was an exploratory test of the method and developed in connection with the revision of WHO *Safe Abortion: Technical and Policy Guidance for Health Systems*. The WHO process for developing guidelines includes a rigorous literature review. IDEAL was developed and tested to help reviewers identify and draw on existing data to explore questions of health effects of abortion law, by identifying potentially important legal questions and pointing to examples of studies that addressed them. The study itself did not aim to identify all relevant research, or to select or classify examples based on rigor. References to specific research studies in this supplement are exemplary, rather than exhaustive or critical.

Finally, we note that the causal models we created do not explicitly include travel to a more permissive jurisdiction as a response to legal restrictions in a pregnant person's home country, or province. This alternative for accessing services may arise anywhere legal restrictions hamper local abortion access, and the phenomenon has been studied in many different legal and regional contexts.[2] Travel to visit an abortion provider, including in another jurisdiction, can be a source of higher costs and delayed healthcare, which are common outcomes covered in the causal models presented.

---

[1] For purposes of this study, laws on third-party authorization for abortion were sub-divided into three categories: parental involvement laws; spousal consent laws; and judicial and police authorization in cases of sexual assault.

[2] For example, Barr-Walker, J., Jayaweera, R. T., Ramirez, A. M., & Gerdts, C. (2019). Experiences of women who travel for abortion: A mixed methods systematic review. PloS one, 14(4), e0209991. https://doi.org/10.1371/journal.pone.0209991; Norris, A. H., Chakraborty, P., Lang, K., Hood, R. B., Hayford, S. R., Keder, L., Bessett, D., Smith, M. H., Hill, B. J., Broscoe, M., Norwood, C. and McGowan, M. L. (2020) Abortion Access in Ohio's Changing Legislative Context, 2010–2018. American Journal of Public Health, 110, 1228-1234. 10.2105/ajph.2020.305706.

Causal Models of Abortion Law

I. Parental Involvement in Minors' Abortion

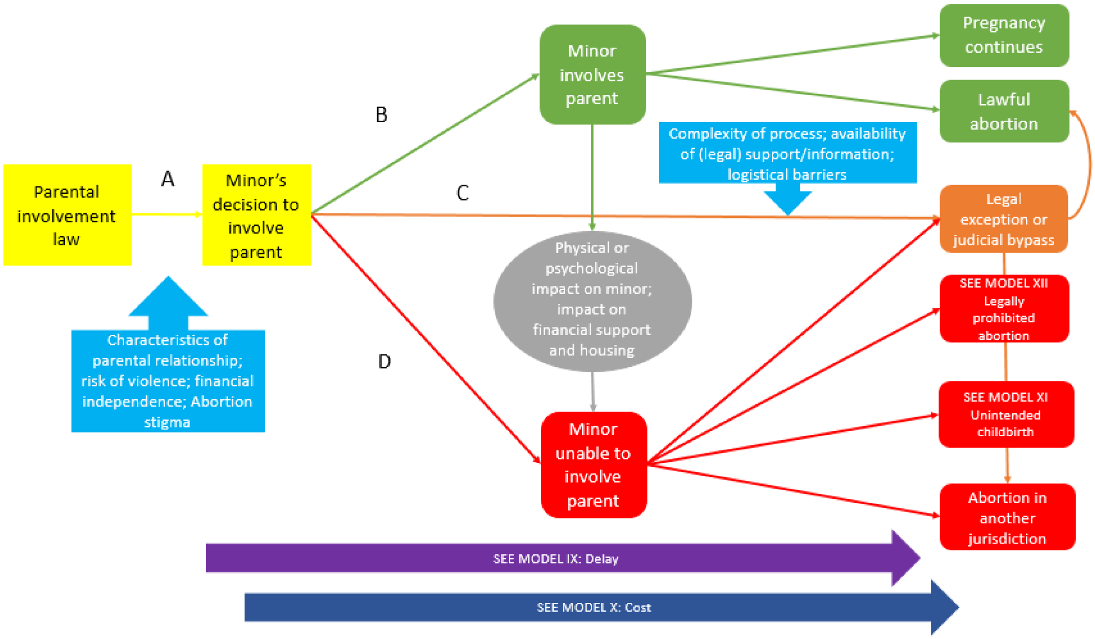

Figure I. Parental Involvement for Minors' Abortion: Causal Logic Model

This model depicts causal pathways related to a parental involvement law. Parental involvement laws require a minor seeking abortion to notify their parents and/or obtain their consent prior to receiving an abortion. These laws may also contain a legal waiver or judicial bypass process that allows them to obtain an abortion without meeting the parental notification or consent requirement.

Pathway A (*yellow*) depicts the mediating effects of contextual social, economic, and relationship factors on a pregnant individual's compliance with a parental notification law. Mediating factors on a minor's decision to involve their parent or not include characteristics of the parental relationship, involvement of a partner, financial ability to seek services, and/or abortion stigma.

Pathway B (*green*) depicts a minor notifying a parent of their desire for abortion, which can clear the minor's path to obtaining an abortion or lead to a decision to proceed with the pregnancy. The model depicts the impact of parental involvement on the health and socioeconomic well-being of the minor, which may produce intrafamilial conflict and other negative consequences for the minor. Such conflict may lead to the minor seeking a legally prohibited abortion or judicial authorization where available, or resulting in unintended childbirth. Proponents of parental notification laws have proposed that informing parents may lead the minor to continue with the pregnancy with positive health and/or social outcomes.

Pathway C (*orange*) represents a minor's decision to pursue legal alternatives to parental consent or notification, such as going to court for judicial approval of an abortion. Accessibility of this option is mediated by the complexity of the alternative process and availability of legal or other assistance

Burris S, *et al. BMJ Global Health* 2021; 6:e005120. doi: 10.1136/bmjgh-2021-005120

services, as well as logistical barriers. Some minors may be unable to complete the process, shifting to the parental involvement or non-compliance pathways. Should the alternative procedure not lead to a lawful abortion, the minor may give birth, obtain a legally prohibited abortion or shift to the parental involvement pathway.[3]

Pathway D (*red*) represents the minor's non-compliance with the parental involvement law, leading to an unintended birth or a legally prohibited abortion. The minor may alternatively choose to travel to another jurisdiction without legal restrictions to obtain an abortion.

All pathways could result in delayed health care and increased costs associated with the law (see Models IX and X).

Table I reports research we identified through the IDEAL process that provides findings on these processes and/or outcomes.

Table I. Parental Involvement for Minors' Abortion: Examples of Research on Identified Causal Pathways

Causal Process	Intermediary or Primary Outcome(s)	Selection of Relevant Studies
Law interacts with character of parental relationship and other contextual factors to produce minor's decision to disclose or avoid parental notification (*Pathway A*)	Minor chooses to involve parent(s) in compliance with legal requirements, or decides to seek alternate authorization or avoid compliance	• Henshaw SK, Kost K. Parental involvement in minors' abortion decisions. *Fam Plann Perspect* 1992 Sep-Oct;24(5):196-207, 213.<br>• Hasselbacher LA, Dekleva A, Tristan S, Gilliam ML. Factors influencing parental involvement among minors seeking an abortion: a qualitative study. *Am J Public Health* 2014;104(11):2207–2211.<br>• Coleman-Minahan K, Stevenson AJ, Obront E, and Hays S. Adolescents Obtaining Abortion Without Parental Consent: Their Reasons and Experiences of Social Support. *Perspectives on Sexual and Reproductive Health* 2020, 52(1):TK. doi:10.1363/psrh.12132

[3] The judicial bypass process in U.S. law and its health effects have been investigated in peer reviewed research. See, for example, Janiak E, Fulcher IR, Cottrill AA, et al. Massachusetts' Parental Consent Law and Procedural Timing Among Adolescents Undergoing Abortion. Obstetrics and Gynecology 2019;133(5):978-986. doi:10.1097/AOG.0000000000003190; Altindag O, Joyce T. Judicial Bypass for Minors Seeking Abortions in Arkansas Versus Other States. American Journal of Public Health 2017;107(8):1266-1271. doi:10.2105/AJPH.2017.303822; Coleman-Minahan K, Stevenson AJ, Obront E, Hays S. Young Women's Experiences Obtaining Judicial Bypass for Abortion in Texas. The Journal of Adolescent Health: Official Publication of the Society for Adolescent Medicine 2019;64(1):20-25. doi:10.1016/j.jadohealth.2018.07.017.

Causal Process	Intermediary or Primary Outcome(s)	Selection of Relevant Studies
Minor involves parent in abortion decision (*Pathway B*)	Parents are supportive of minor's decision to proceed to abortion or childbirth	• Stidham-Hall K, Moreau C, Trussell J. Patterns and correlates of parental and formal sexual and reproductive health communication for adolescent women in the United States, 2002-2008. *The Journal of Adolescent Health: Official Publication of the Society for Adolescent Medicine* 2012;50(4):410-413. doi:10.1016/j.jadohealth.2011.06.007
	Parental opposition or lack of support influences minor's financial or emotional well-being	• Ralph L, et al. The Role of Parents and Partners in Minors' Decisions to Have an Abortion and Anticipated Coping After Abortion. *Journal of Adolescent Health* 2014;54(4):428-434.<br>• Henshaw SK, Kost K, Parental involvement in minors' abortion decisions. *Fam Plann Perspect* 1992;24(5):196-207, 213.
Minor seeks judicial bypass or legal exception (*Pathway C*)	Process of seeking judicial bypass is mediated by availability of legal resources and logistical barriers, in some cases leading to delayed or inaccessible abortion	*See Model IX for Delay*<br>*See Model X for Cost*
Minor does not comply with legal requirement (*Pathway D*)	Unintended childbirth<br>Prohibited abortion<br>Abortion in another jurisdiction	*See Model XI for Unintended Childbirth*<br>*See Model XII for Legally Prohibited Abortion*<br>• Myers C, Ladd D. Did parental involvement laws grow teeth? The effects of state restrictions on minors' access to abortion. J Health Econ. 2020;71:102302. doi:10.1016/j.jhealeco.2020.102302

II. Spousal Consent for Abortion

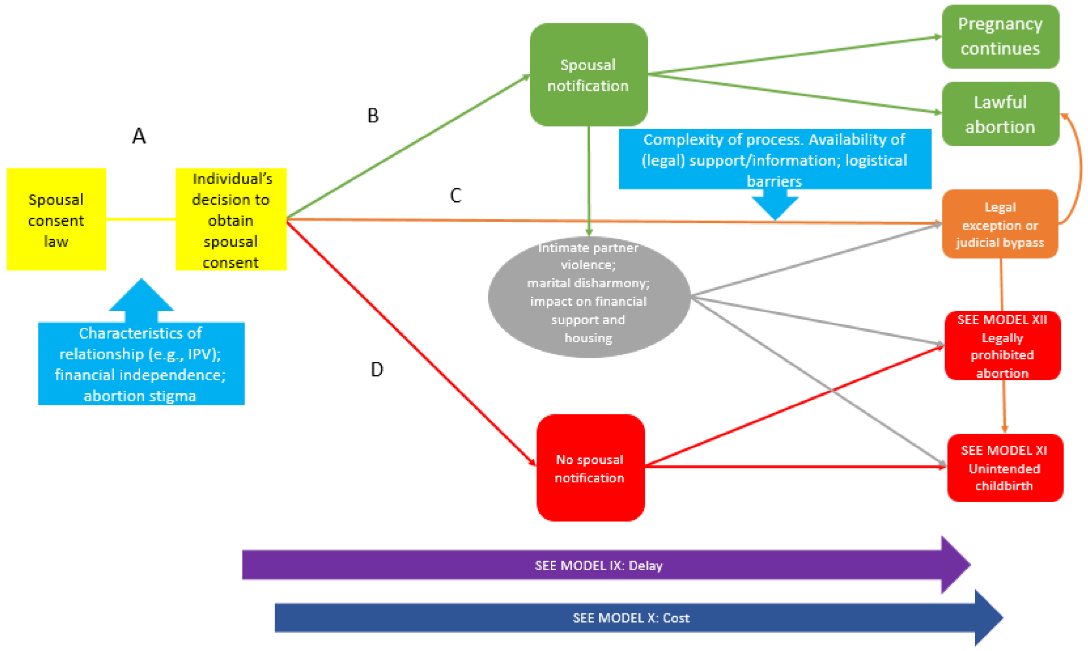

Figure II. Spousal Consent for Abortion: Causal Logic Model

This model depicts causal pathways related to a spousal consent law. Spousal consent laws require a pregnant individual to obtain the consent of a spouse prior to receiving an abortion. The law may provide exceptions to the spousal consent requirement in certain circumstances.

Pathway A (*yellow*) depicts the mediating effects of contextual social, economic and relationship factors on a pregnant individual's compliance with a spousal notification law.

Pathway B (*green*) depicts a pregnant person notifying a spouse of their desire for abortion, which can clear the pregnant person's path to obtaining an abortion or lead to a decision to proceed with the pregnancy. The model depicts the impact of spousal involvement on the health and socioeconomic well-being of the pregnant person, which may produce intrafamilial conflict and other negative consequences. Such conflict may lead to the pregnant person seeking a legally prohibited abortion or a legal option to avoid spousal consent or notification where available, or an unintended childbirth.

Pathway C (*orange*) represents a pregnant person's decision to pursue legal alternatives to spousal consent or notification. Accessibility of this option is mediated by the nature of the qualifying circumstances, the complexity of any alternative process and availability of legal or other assistance services, as well as logistical barriers. Some pregnant persons may be unable to qualify for or attain an exemption, shifting them to the spousal involvement or non-compliance pathways. Should the exception process not lead to a lawful abortion, the pregnant person may give birth, obtain a legally prohibited abortion or shift to the spousal involvement pathway. The pregnant person may also choose to travel to another jurisdiction without legal restrictions to obtain an abortion (not shown).

Pathway D (*red*) represents the pregnant person's non-compliance with the spousal involvement law, leading to an unintended birth or a legally prohibited abortion. Mediating factors on a pregnant person's decision to involve their parent or not can depend on characteristics of the spousal relationship, financial ability to seek services, and/or abortion stigma.

All pathways could result in delayed health care and increased costs associated with the law.

Table II reports research we identified through the IDEAL process that provides findings on these processes and/or outcomes.

Table II. Spousal Consent for Abortion: Examples of Research on Identified Causal Pathways

Causal Process	Intermediate or Primary Outcome(s)	Examples of Relevant Studies
Law interacts with character of spousal relationship and other contextual factors to produce pregnant individual's decision to seek or avoid spousal consent (*Pathway A*)	Pregnant individual chooses to involve spouse in compliance with legal requirements, or decides to seek alternate authorization or avoid compliance	• Colarossi L, & Dean G. Partner violence and abortion characteristics. *Women & health* 2014;54(3):177–193. https://doi.org/10.1080/03630242.2014.883662<br>• Chibber KS, et al. The Role of Intimate Partners in Women's Reasons for Seeking Abortion, *Women's Health Issues* January–February 2014; 24(1):e131-e138<br>• Gupte M, Bandewar S, Pisal H. Women's perspectives on the quality of general and reproductive health care: evidence from rural Maharashtra, *Improving Quality of Care in India's Family Welfare Programme* 1999.<br>• Astbury-Ward E, Parry O, & Carnwell R. Stigma, abortion, and disclosure--findings from a qualitative study. *The journal of sexual medicine* 2014;9(12):3137–3147. https://doi.org/10.1111/j.1743-6109.2011.02604.x
Pregnant individual seeks spouse's consent for abortion (*Pathway B*)	Spouse is supportive of decision to proceed to abortion or childbirth	• Altshuler, Nguyen et al., Male Partners' Involvement in Abortion Care: A Mixed-Methods Systematic Review, *Perspect Sex Reprod Health* 2016 Dec; 48:209-219.

Causal Process	Intermediate or Primary Outcome(s)	Examples of Relevant Studies
	Spouse is not supportive of an abortion decision, leading to risk of conflict or physical or economic harm or unintended childbirth	• Woo J, Fine P, Goetzl L. Abortion disclosure and the association with domestic violence. *Obstet Gynecol*. 2005 Jun;105(6):1329-34.<br>• Stephenson R, et al. Domestic Violence and Abortion Among Rural Women in Four Indian States, Violence *Against Women* 2016;22: 1642<br>• Hall M, Chappell LC, Parnell BL, Seed PT, & Bewley S. Associations between intimate partner violence and termination of pregnancy: a systematic review and meta-analysis. *PLoS medicine* 2014;11(1):e1001581. https://doi.org/10.1371/journal.pmed.1001581
Pregnant individual seeks legal alternative to spousal notification/consent (*Pathway C*)	Process of seeking legal alternative is mediated by availability of legal resources and logistical barriers, in some cases leading to delayed or inaccessible abortion	*See Model IX for Delay*<br>*See Model X for Cost*
Pregnant individual avoids spousal notification (*Pathway D*)	Pregnant individual obtains a legally prohibited abortion<br>Pregnant individual is deterred from seeking abortion and continues the pregnancy	*See Model XII for Legally Prohibited Abortion*<br>*See Model XI for Unintended Childbirth*

III. Additional Authorization in Cases of Sexual Assault

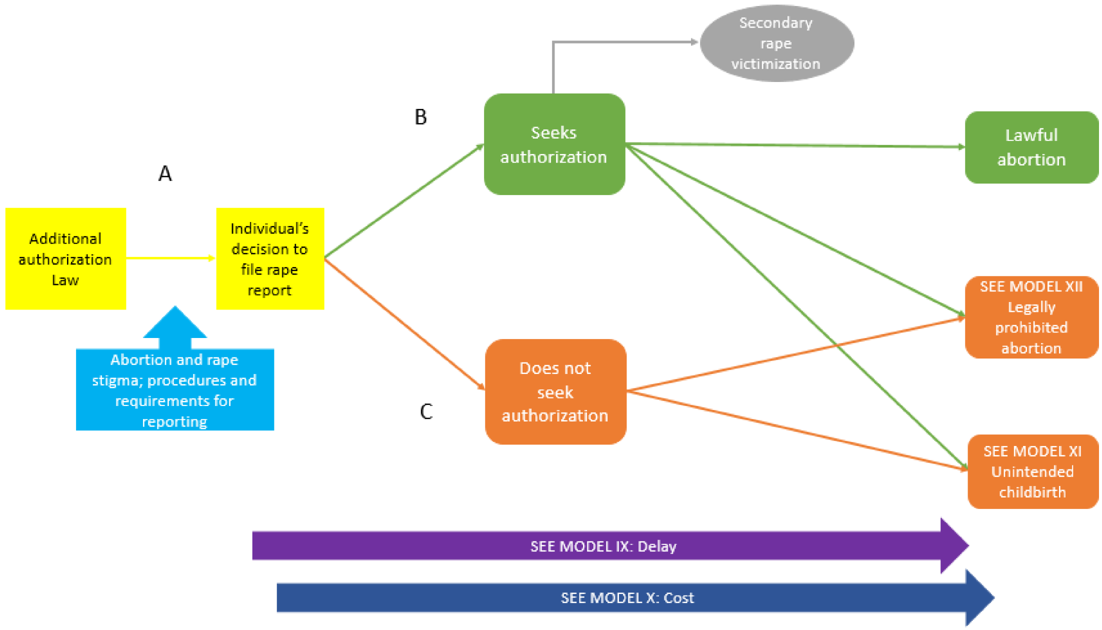

Figure III. Additional Authorization in Cases of Sexual Assault: Causal Logic Model

This model depicts pathways related to laws that allow an otherwise prohibited abortion in a case of sexual assault, provided the person seeking the abortion obtains judicial or police authorization.

Pathway A (*yellow*) depicts the mediating effects of contextual social, economic and relationship factors on a pregnant individual's decision to seek authorization required by law.

Pathway B (*green*) depicts pursuit of the process for additional authorization to obtain a lawful abortion. The process of obtaining authorization can lead to further harm from secondary victimization of sexual violence through invasive questioning and medical tests to verify legal exceptions. Procedural roadblocks may result in delaying the abortion beyond legal gestational limits, foreclosing the possibility of lawful abortion. This pathway also shows that compliance with the procedural requirements does not guarantee access to a safe, legal abortion. Police may refuse to find that a rape has occurred, or a medical board may find that statutory criteria are not satisfied, leaving the pregnant individual to seek an abortion outside legal parameters or lead to unintended childbirth. [4]

Pathway C (*orange*) represents a pregnant individual's noncompliance with an additional authorization requirement. Factors that influence an individual's willingness or ability to obtain authorization may include structural barriers, sexual assault and abortion stigma, and provider attitudes. The individual may proceed to an abortion outside of legal parameters or experience unintended childbirth.

[4] This example does not contemplate approval by hospital or government-led committees, though the model could be applicable to those means of third-party authorization, and does not reflect abortion permissible for grounds other than sexual assault, such as threat to the pregnant individual's life.

Each of these pathways may also contribute to increases in delayed care or costs. Travel to another jurisdiction to avoid the rule (not shown) can also increase costs or cause delay.

Table III reports research we identified through the IDEAL process that provides findings on these processes and/or outcomes.

Table III. Additional Authorization in Cases of Sexual Assault: Examples of Research on Identified Causal Pathways

Causal Process	Intermediary or Primary Outcome(s)	Selection of Relevant Studies
Law interacts with contextual factors to influence a pregnant individual's decision to seek or avoid third party authorization (*Pathway A*)	Pregnant individual decides to seek authorization or to proceed without seeking legal abortion	• Lara D, García S, Ortiz O, Yam EA. Challenges accessing legal abortion after rape in Mexico City. *Gac Med Mex* 2006 Sep-Oct;142 Suppl 2:85-9<br>• Blake M, Drezett J, et al. Factors associated with the delay in seeking legal abortion for pregnancy resulting from rape. *International Archives Of Medicine* 2015;8. doi:10.3823/1628.<br>• Silva M, Billings DL, García, SG, & Lara D. Physicians' agreement with and willingness to provide abortion services in the case of pregnancy from rape in Mexico. *Contraception* 2009;79(1):56–64. https://doi.org/10.1016/j.contraception.2008.07.016<br>• Machado CL, Fernandes AM, Osis MJ., & Makuch MY. Gravidez após violência sexual: vivências de mulheres em busca da interrupção legal [Rape-related pregnancy in Brazil: the experience of women seeking legal abortion]. *Cadernos de saude publica* 2015;31(2):345–353. https://doi.org/10.1590/0102-311x00051714<br>• Hodoglugil S, et al . Making Abortion Safer in Rwanda: Operationalization of the Penal Code of 2012 to Expand Legal Exemptions and Challenges. Afr J Reprod Health 2017 Mar;21(1):82-92.

Causal Process	Intermediary or Primary Outcome(s)	Selection of Relevant Studies
Pregnant individual complies with additional authorization requirement (*Pathway B*)	Individual experiences secondary rape victimization	• Campbell R, Raja S. Secondary victimization of rape victims: insights from mental health professionals who treat survivors of violence. *Violence and victims* 1999;14(3):261–275.<br>• Maier SL."I have heard horrible stories . . .": rape victim advocates' perceptions of the revictimization of rape victims by the police and medical system. *Violence against women* 2008; 14(7):786–808. https://doi.org/10.1177/1077801208320245<br>• Machado CL, Fernandes AM, Osis MJ, & Makuch MY. Gravidez após violência sexual: vivências de mulheres em busca da interrupção legal [Rape-related pregnancy in Brazil: the experience of women seeking legal abortion]. *Cadernos de saude publica* 2015;31(2):345–353. https://doi.org/10.1590/0102-311x00051714
	Individual obtains lawful abortion	• Raymond EG, & Grimes DA. *The comparative safety of legal induced abortion and childbirth in the United States*. Obstetrics and gynecology 2012;119(2 Pt 1):215–219. https://doi.org/10.1097/AOG.0b013e31823fe923
	Denial of legal authorization leads to legally prohibited abortion or unintended childbirth	*See Model XII for Legally Prohibited Abortion*<br>*See Model XI for Unintended Childbirth*
Pregnant individual does not comply with additional authorization requirement (*Pathway C*)	Legally prohibited abortion or unintended childbirth	*See Table XII for Legally Prohibited Abortion*<br>*See Model XI for Unintended Childbirth*

IV. Gestational Limits

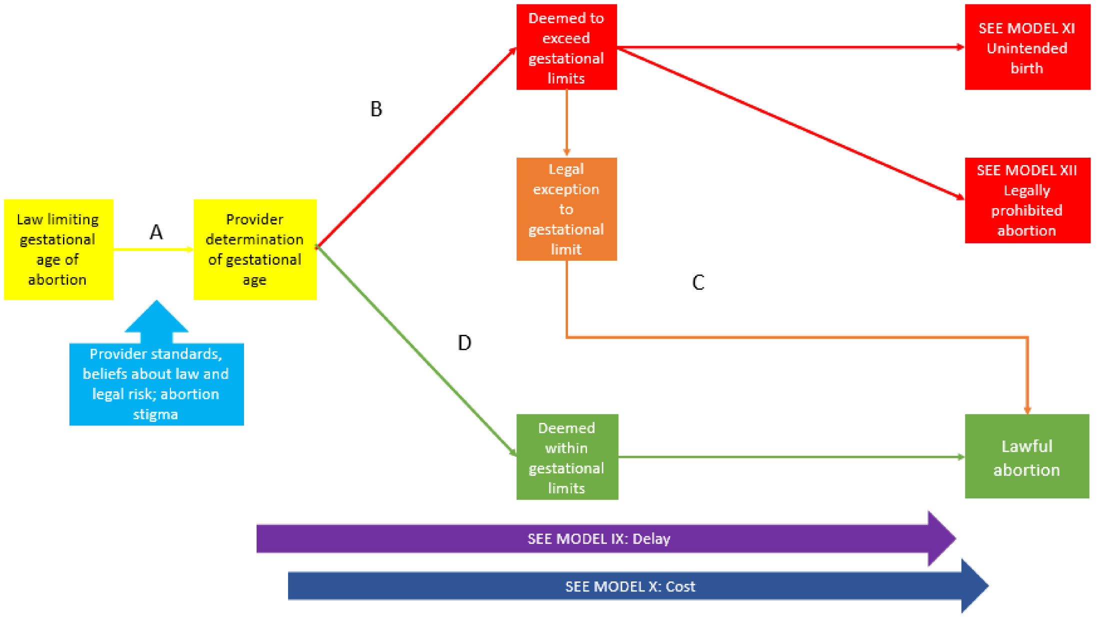

Figure IV. Gestational Limits: Causal Logic Model

This model depicts causal pathways related to a law imposing a gestational age limit that regulates when in the course of a pregnancy an abortion can occur. The law may include legal exceptions that provide grounds for obtaining abortion beyond the gestational limit in certain cases, such as rape or where the pregnant individual's life is endangered.

Pathway A (*yellow*) depicts the mediating effects of clinical standards, provider beliefs about law and the legal risk of providing an abortion given uncertainty about gestational age, and social attitudes towards abortion on the provider's determination of gestational age. Provider standards are influenced by knowledge, attitudes and beliefs about the gestational age limit law, which may have a chilling effect such that providers become unwilling to perform abortions within the legal gestational age range.

Pathway B (*red*) depicts a pregnant individual whose pregnancy is determined to exceed the gestational age for abortion set by law. After exceeding the gestational age limit, the individual may have an unwanted or unintended childbirth or may seek an abortion outside legal parameters.

Pathway C *(orange)* depicts the pathway of a pregnant individual who exceeds the gestational age set by law but qualifies for an exception based on legal grounds and obtains a lawful abortion. Some studies have investigated the implementation and effects of laws creating exceptions to abortion prohibitions.[5]

[5] See, for example, Küng, S. A., Darney, B. G., Saavedra-Avendaño, B., Lohr, P. A. and Gil, L. (2018) Access to abortion under the heath exception: a comparative analysis in three countries. Reproductive health, 15, 107. 10.1186/s12978-018-0548-x.

Pathway D *(green)* depicts the pathway of a pregnant individual seeking an abortion within the gestational age limit set by law and obtaining a lawful abortion.

Pathways B and C may contribute to delayed care or increased costs even for those who obtain a legal abortion. Travel to another jurisdiction to avoid the rule (not shown) can also increase costs or cause delay.

Table IV reports research we identified through the IDEAL process that provides findings on these processes and/or outcomes.

Table IV. Gestational Limits: Examples of Research on Identified Causal Pathways

Causal Process	Intermediary or Primary Outcome(s)	Selection of Relevant Studies
Legal gestational limits interact with diagnostic practices and provider tolerance of legal risk to produce a finding that the pregnancy is past the limit (*Pathway A*)	Provider makes a determination of gestational age	• Black, K. I., Douglas, H., & de Costa, C. (2015). Women's access to abortion after 20 weeks' gestation for fetal chromosomal abnormalities: Views and experiences of doctors in New South Wales and Queensland. *The Australian & New Zealand journal of obstetrics & gynaecology*, 55(2), 144–148. https://doi.org/10.1111/ajo.12305
Pregnancy deemed to exceed gestational limits and does not fall within legal exception *(Pathway B)*	Unintended birth or legally prohibited abortion	*See Model XII for Legally Prohibited Abortion*<br>*See Model XI for Unintended Childbirth*
Legal exception to gestational limit available (*Pathway C*)	Pregnant individual obtains safe legal abortion	*See Model IX for Delay*<br>*See Model X for Cost*
Individual seeking abortion is deemed to be within gestational limit (*Pathway D*)	Pregnant individual obtains safe legal abortion	

V. Mandatory Waiting Periods

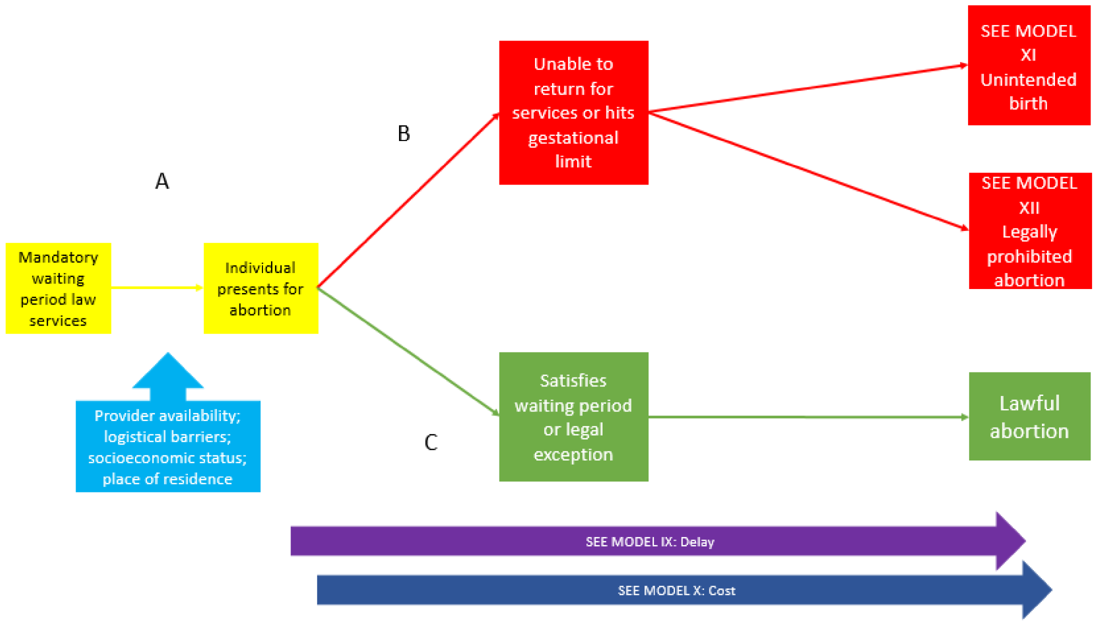

Figure V. Mandatory Waiting Periods: Causal Logic Model

This model depicts causal pathways related to a mandatory waiting period requirement. Mandatory delay or waiting period laws require an individual seeking abortion to wait for a prescribed period of time (e.g. 48 hours) between the initial visit and receiving an abortion. The law may include exceptions to the waiting period such as in cases of medical emergency.

The health and other effects of waiting period requirements have been relatively well-studied. Studies have documented legal effects including higher cost, the utilization of medication abortion, and abortion being delayed past gestational limits.[6] Table V does not include references to research explicitly studying legal effects.

Pathway A (*yellow*) depicts the mediating effects of provider availability and the socio-economic status and residence location of the pregnant individual on the process of finding a provider and presenting for

---

[6] For example, see Karasek, D., Roberts, S. C., & Weitz, T. A. (2016). Abortion Patients Experience and Perceptions of Waiting Periods: Survey Evidence before Arizona's Two-visit 24-hour Mandatory Waiting Period Law. Womens Health Issues, 26(1), 60-66. doi:10.1016/j.whi.2015.10.004; Roberts, S. C., Turok, D. K., Belusa, E., Combellick, S., & Upadhyay, U. D. (2016). Utah's 72-Hour Waiting Period for Abortion: Experiences Among a Clinic-Based Sample of Women. Perspectives on sexual and reproductive health, 48(4), 179–187. https://doi.org/10.1363/48e8216; Althaus, F.A. and Henshaw, S.K. (1994). The Effects of Mandatory Delay Laws on Abortion Patients and Providers. Family Planning Perspectives, 26(5), 228. doi:10.2307/2135944; Joyce, T., & Kaestner, R. (2000). The impact of Mississippi's mandatory delay law on the timing of abortion. Family planning perspectives, 32(1), 4–13. For a review of U.S. studies, see Joyce, T. J., Henshaw, S. K., Dennis, A., Finer, L. B. and Blanchard, K. (2009) The Impact of State Mandatory Counseling and Waiting Period Laws on Abortion: A Literature Review. Guttmacher Institute, New York.

abortion services, which triggers the waiting period. The time required for this process will determine how close the individual is to a gestational age limit.

Pathway *(red)* depicts the pathway of a pregnant individual who is unable to return for care at the expiration of the waiting period requirement due to logistical barriers such as finances and childcare, and accessibility of clinics or providers. It also captures the case of compliance with the waiting period and related delays leading to the pregnant individual reaching a gestational limit. As a result of inability to comply, the individual may have an unwanted or unintended childbirth, or seek an abortion outside legal parameters. (Not shown: the individual who reaches the gestational limit may qualify for an exception and be able to secure a legal abortion; see Model IV.)

Pathway C *(green)* depicts the pathway of a pregnant individual who is able to return for service after the waiting period requirement and obtain a lawful abortion. This includes individuals who qualify for an exemption, such as medical emergency. Compliance with the law may contribute to increased costs and delay in obtaining care.

Both of these pathways may also contribute to increases in delayed care or costs. Travel to another jurisdiction to avoid the rule (not shown) can also increase costs or cause delay.

Table V reports research we identified through the IDEAL process that provides findings on these processes and/or outcomes.

Table V. Mandatory Waiting Periods: Examples of Research on Identified Causal Pathways

Causal Process	Intermediary or Primary Outcome(s)	Selection of Relevant Studies
The impact of mandatory waiting period laws is mediated by other individual and contextual factors influencing the gestational point at which the pregnant individual presents for an abortion (*Pathway A*)	Pregnant individual presents to a provider for an abortion	• Ushie, B. A., Izugbara, C. O., Mutua, M. M. and Kabiru, C. W. (2018) Timing of abortion among adolescent and young women presenting for post-abortion care in Kenya: a cross-sectional analysis of nationally-representative data. BMC Women's Health, 18, 41. 10.1186/s12905-018-0521-4.<br>• Pinter, B., Aubeny, E., Bartfai, G., Loeber, O., Ozalp, S., & Webb, A. (2005). Accessibility and availability of abortion in six European countries. The European journal of contraception & reproductive health care: the official journal of the European Society of Contraception, 10(1), 51–58. https://doi.org/10.1080/13625180500035231

Causal Process	Intermediary or Primary Outcome(s)	Selection of Relevant Studies
Pregnant individual is unable to comply with waiting period or reaches a gestational limit during the wait (*Pathway B*)	Unintended childbirth or legally prohibited abortion	*See Model XI for Unintended Childbirth*<br>*See Model XII for Legally Prohibited Abortion*
Pregnant individual satisfies or is exempt from the waiting period and remains eligible for a legal abortion (*Pathway C*)	Safe, legal abortion<br>Delayed abortion care<br>Increased costs due to compliance or delayed care	*See Model IX for Delay*<br>*See Model X for Cost*

VI. Provider Restrictions

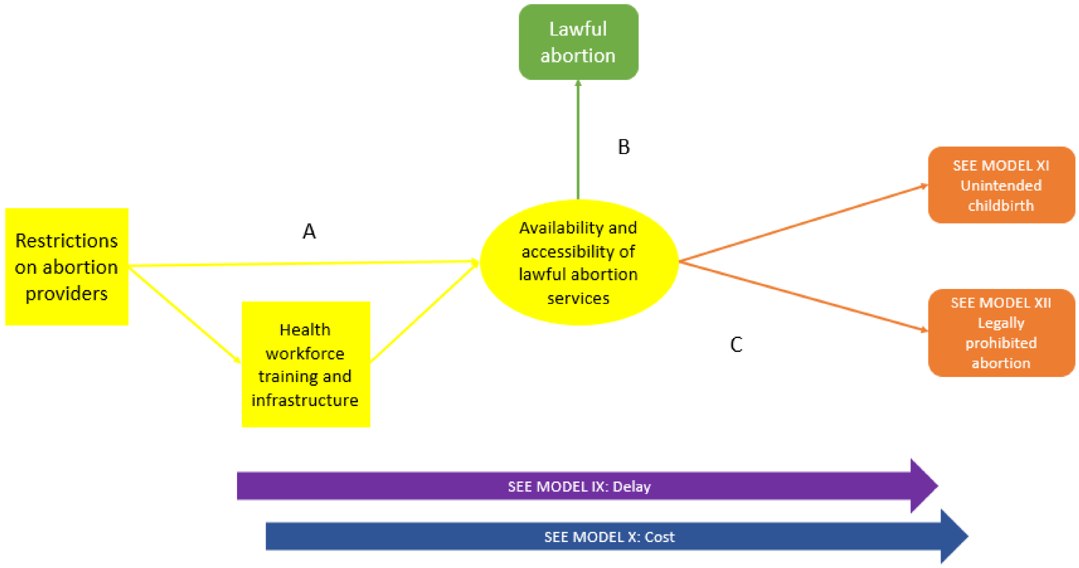

Figure VI. Provider Restrictions: Causal Logic Model

WHO guidelines advise that a wide range of medical professionals, including primary-care physicians, physician assistants, and nurses can provide safe and effective abortion services in a variety of settings.[7] This model depicts possible health-system effects of laws that restrict the types of health care licensees who may provide an abortion (e.g. physicians only), require special certifications and trainings, or limit settings where abortion may be provided (e.g. a state hospital).

Pathway A (*yellow*) depicts the influence of these laws on the availability and accessibility of availability of abortion providers directly and as a function of law's influence on training and the organization of abortion services. The effect may also be mediated by the availability of self-managed abortion (not shown).[8]

---

[7] Researchers of abortion law have examined this issue. See, e.g., Joffe C, Yanow S. Advanced practice clinicians as abortion providers: current developments in the United States. Reprod Health Matters. 2004;12(Suppl):198–206. doi: 10.1016/S0968-8080(04)24008-3; Berer M. (2009). Provision of abortion by mid-level providers: international policy, practice and perspectives. Bulletin of the World Health Organization, 87(1), 58–63. https://doi-org.libproxy.temple.edu/10.2471/blt.07.050138; Battistelli, M. F., Magnusson, S., Biggs, M. A., & Freedman, L. (2018). Expanding the Abortion Provider Workforce: A Qualitative Study of Organizations Implementing a New California Policy. Perspectives on sexual and reproductive health, 50(1), 33–39. https://doi.org/10.1363/psrh.12051; Battistelli, M. F., Magnusson, S., Biggs, M. A. and Freedman, L. (2018) Expanding the Abortion Provider Workforce: A Qualitative Study of Organizations Implementing a New California Policy. Perspect Sex Reprod Health, 50, 33-39. https://doi.org/10.1363/psrh.12051.

[8] See, e.g., Moseson, H., Herold, S., Filippa, S., Barr-Walker, J., Baum, S. E., & Gerdts, C. (2020). Self-managed abortion: A systematic scoping review. Best practice & research. Clinical obstetrics & gynaecology, 63, 87–110. https://doi.org/10.1016/j.bpobgyn.2019.08.002.

Pathway B (*green*) depicts the path to lawful abortion for those who are able to obtain abortion services.

Pathway C (*orange*) depicts the results for individuals unable to access lawful abortion as a result of the decrease in abortion providers. The lack of available abortion providers may result in abortion obtained outside legal parameters or unintended childbirth.

Each of these pathways may also contribute to increases in delayed care or costs. Travel to another jurisdiction to access services (not shown) can also increase costs or cause delay.

Table VI reports research we identified through the IDEAL process that provides findings on these processes and/or outcomes.

Table VI. Provider Restrictions: Examples of Research on Identified Causal Pathways

Causal Process	Intermediary or Primary Outcome(s)	Selection of Relevant Studies
Law restricting availability of abortion providers interact with health workforce training and infrastructure (*Pathway A*)	Availability and accessibility of abortion services	• Grimes DA. Clinicians who provide abortions: the thinning ranks. *Obstet Gynecol*. 1992 Oct;80(4):719-23. PMID: 1407901.<br>• Jones, R. K., & Jerman, J. (2014). Abortion incidence and service availability in the United States, 2011. *Perspectives on sexual and reproductive health*, 46(1), 3–14. https://doi.org/10.1363/46e0414<br>• Leslie, D. L., Liu, G., Jones, B. S., & Roberts, S. (2020). Healthcare costs for abortions performed in ambulatory surgery centers vs office-based settings. *American journal of obstetrics and gynecology*, 222(4), 348.e1–348.e9. https://doi.org/10.1016/j.ajog.2019.10.002
Shortage of providers increases cost or causes delay in obtaining lawful abortion (*Pathway B*)	Delayed or more expensive abortion	*See Model IX for Delay*<br>*See Model X for Cost*
Lawful abortion unavailable due to decrease in providers (*Pathway C*)	Unintended childbirth or legally prohibited abortion	*See Table XI for Unintended Childbirth*<br>*See Table XII for Legally Prohibited Abortion*

VII. Criminalization of Abortion

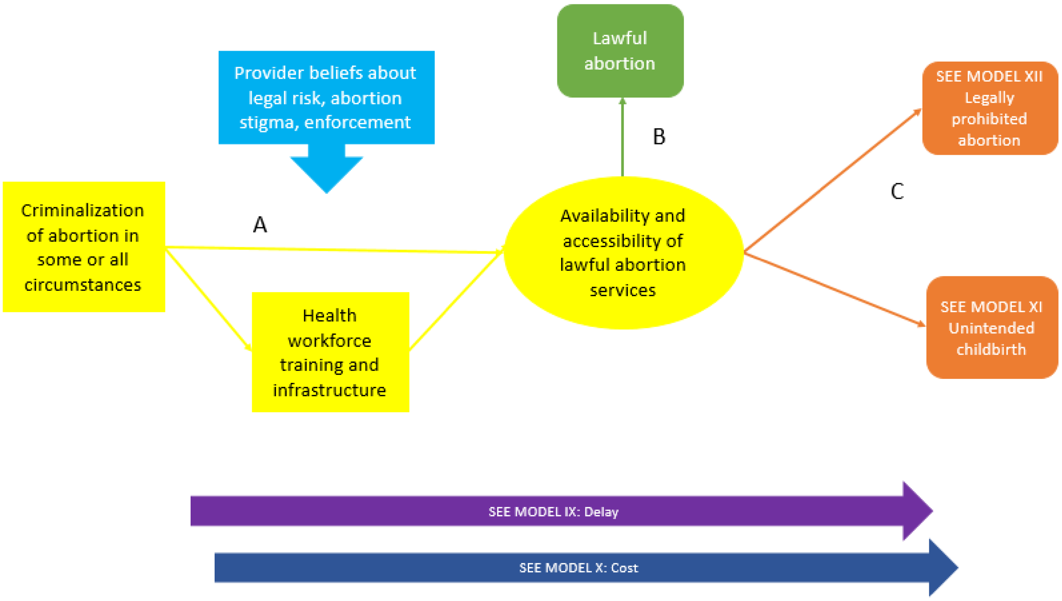

Figure VII. Criminalization of Abortion: Causal Logic Model

"Criminalization of abortion" refers to the enactment of penalties under criminal law for abortion related health services. These may include prohibition of all abortions, or of abortions performed outside of set legal limits, such as gestational age. These laws may also cover self-managed abortion and the prescribing, dispensing, administration or use of medications for abortion. The deterrent effect of criminal sanctions may operate directly on provider willingness to provide services at all, and through a reduction in training and service infrastructure for abortion.

Pathway A (*yellow*) depicts the influence of these laws on the availability and accessibility of availability of abortion providers directly and as a function of law's influence on training and the organization of abortion services. These effects are mediated by providers' perceptions or experience of legal risk, generalized abortion stigma and the degree of enforcement of criminal laws.[9]

Pathway B (*green*) depicts the path to lawful abortion for those who are able to obtain abortion services.

[9] "Criminalization" is related to or can be understood as a possible characteristic of any abortion regulation. There is limited research on the effects of particular criminal penalties as such, but numerous studies examine how the overall abortion legal environment can influence accessibility and availability of services. See, e.g., Norris, A. H., Chakraborty, P., Lang, K., Hood, R. B., Hayford, S. R., Keder, L., Bessett, D., Smith, M. H., Hill, B. J., Broscoe, M., Norwood, C. and McGowan, M. L. (2020) Abortion Access in Ohio's Changing Legislative Context, 2010–2018. American Journal of Public Health, 110, 1228-1234. 10.2105/ajph.2020.305706; McNaughton, H. L., Mitchell, E. M., & Blandon, M. M. (2004). Should doctors be the judges? Ambiguous policies on legal abortion in Nicaragua. Reproductive health matters, 12(24 Suppl), 18–26. https://doi.org/10.1016/s0968-8080(04)24005-8.

Pathway C (*orange*) depicts lack of access to abortion within legal parameters, leading to unintended childbirth or seeking a legally prohibited abortion.

Each of these pathways may also contribute to increases in delayed care or costs. Travel to another jurisdiction to access services (not shown) can also increase costs or cause delay.

Table VII reports research we identified through the IDEAL process that provides findings on these processes and/or outcomes.

Table VII. Criminalization of Abortion: Examples of Research on Identified Causal Pathways

Causal Process	Intermediary or Primary Outcome(s)	Selection of Relevant Studies
Law providing criminal penalties for abortion law infractions interacts with health workforce training and infrastructure, provider perceptions of legal risk, abortion stigma and law enforcement (*Pathway A*)	Availability and accessibility of abortion services	• Summit, A. K., Lague, I., Dettmann, M. and Gold, M. (2020) Barriers to and Enablers of Abortion Provision for Family Physicians Trained in Abortion During Residency. *Perspect Sex Reprod Health*, 52, 151-159. https://doi.org/10.1363/psrh.12154.<br>• Moseson, H., Herold, S., Filippa, S., Barr-Walker, J., Baum, S. E., & Gerdts, C. (2020). Self-managed abortion: A systematic scoping review. *Best practice & research. Clinical obstetrics & gynaecology*, 63, 87–110. https://doi.org/10.1016/j.bpobgyn.2019.08.002<br>• Sorhaindo, A. M., Juárez-Ramírez, C., Díaz Olavarrieta, C., Aldaz, E., Mejía Piñeros, M. C., & Garcia, S. (2014). Qualitative evidence on abortion stigma from Mexico City and five states in Mexico. *Women & health*, 54(7), 622–640. https://doi.org/10.1080/03630242.2014.919983<br>• Hanschmidt, F., Linde, K., Hilbert, A., Riedel-Heller, S. G., & Kersting, A. (2016). Abortion Stigma: A Systematic Review. *Perspectives on sexual and reproductive health*, 48(4), 169–177.

Causal Process	Intermediary or Primary Outcome(s)	Selection of Relevant Studies
Availability and accessibility influences cost or causes delay in obtaining lawful abortion (*Pathway B*)	Delayed or more expensive abortion	*See Model IX for Delay*<br>*See Model X for Cost*
Lack of accessible abortion services leads to inability to obtain a safe, legal abortion *(Pathway C)*	Unintended childbirth or legally prohibited abortion	*See Table XI for Unintended Childbirth*<br>*See Table XII for Legally Prohibited Abortion*

VIII. Conscientious Objection

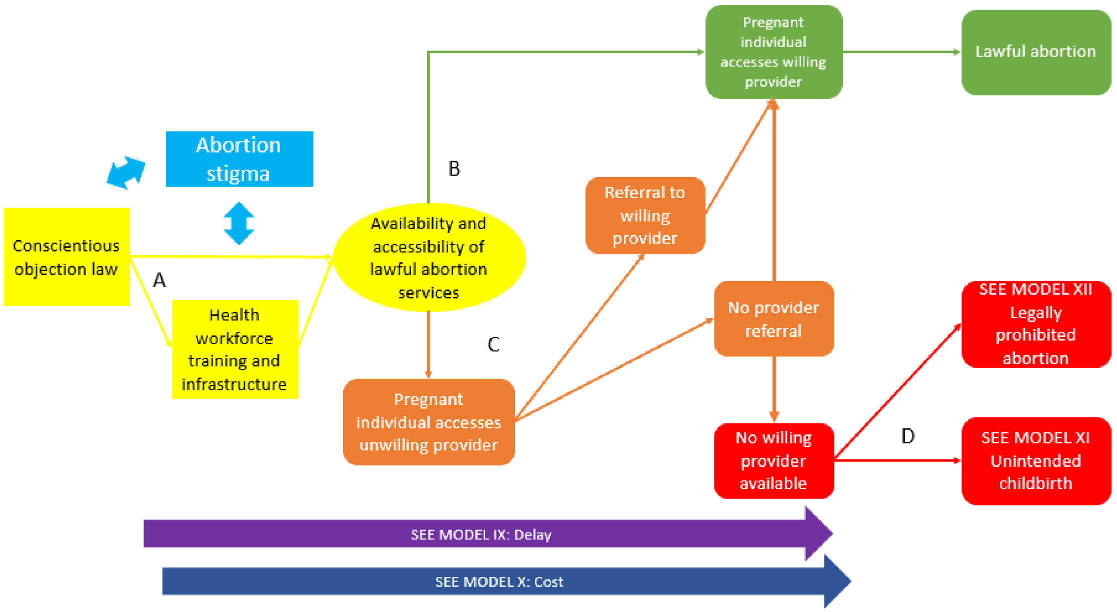

Figure VIII. Conscientious Objection: Causal Logic Model

Conscientious objection laws allow an individual medical provider or facility by policy to refuse to perform an abortion based on personal, moral, or religious beliefs. These laws may or may not require that objecting providers give a referral for abortion or perform abortion in cases of medical emergency.

Pathway A (*yellow*) depicts the influence of these laws on the availability and accessibility of availability of abortion providers directly and as a function of law's influence on training and the organization of abortion services. These effects are mediated by generalized abortion stigma and abortion stigma may both reflect and influence the enactment of such laws.[10]

Pathway B (*green*) depicts the path of a patient who is able to find a willing, and accessible abortion provider, resulting in a lawful abortion. Where the availability and accessibility of willing abortion providers decrease, Pathway B may lead to an increase in cost and delay in obtaining abortion.

[10] Some empirical legal research has examined the implementation and impact of these laws. See, e.g., E. Freeman, E. Coast. Conscientious objection to abortion: Zambian healthcare practitioners' beliefs and practices. Soc Sci Med, 221 (2019), pp. 106-114, 10.1016/j.socscimed.2018.12.018; Bo, M., Zotti, C. M., & Charrier, L. (2015). Conscientious objection and waiting time for voluntary abortion in Italy. The European journal of contraception & reproductive health care: the official journal of the European Society of Contraception, 20(4), 272–282. https://doi.org/10.3109/13625187.2014.990089; Autorino, T., Mattioli, F., & Mencarini, L. (2020) The impact of gynecologists' conscientious objection on abortion access. Social science research, 87, 102403. https://doi.org/10.1016/j.ssresearch.2020.102403; Chavkin, W., Swerdlow, L. and Fifield, J. (2017) Regulation of Conscientious Objection to Abortion: An International Comparative Multiple-Case Study. Health and Human Rights, 19, 55-68.

Pathway C (*orange*) depicts possible causal chains arising from an objecting medical provider or institution. Legal provisions may or may not require the objector to provide a referral to a willing abortion provider. Pregnant individuals who eventually find a non-objecting provider may suffer emotional harm and stigmatization, experience delay in obtaining an abortion, and/or incur higher costs due to the need to travel or manage other logistical challenges.[11]

Pathway D (*red*) depicts a pregnant individual who is unable to find a willing provider, particularly in an environment with restricted abortion access. The pathway may result from the general lack of willing providers due to conscientious objection, or inability to find a willing provider at all or within applicable gestational limits. Pregnant individuals unable to access lawful abortion services in time may be faced with unintended childbirth or obtaining abortion outside legal parameters, with delay and cost compounding the effects of provider refusal.[12] These may have negative health and socioeconomic outcomes, as well as health systems costs.

Travel to another jurisdiction to access services (not shown) can also increase costs or cause delay.

Table VIII reports research we identified through the IDEAL process that provides findings on these processes and/or outcomes.

Table VIII. Conscientious Objection: Examples of Research on Identified Causal Pathways

Causal Process	Intermediary or Primary Outcome(s)	Selection of Relevant Studies
Conscientious objection law, abortion stigma, and provider training infrastructure interact (*Pathway A*)	Availability and accessibility of providers	• Turner, K. L., Pearson, E., George, A., & Andersen, K. L. (2018). Values clarification workshops to improve abortion knowledge, attitudes and intentions: a pre-post assessment in 12 countries. *Reproductive health*, 15(1), 40. https://doi.org/10.1186/s12978-018-0480-0
Availability and accessibility influences cost or causes	Delayed or more expensive abortion	*See Model IX for Delay*<br>*See Model X for Cost*

[11] Provider referral and other behavior and attitudes related to conscientious objection law is investigated in numerous studies cited in footnote 11 and in Awoonor-Williams, J. K., Baffoe, P., Aboba, M., Ayivor, P., Nartey, H., Felker, B., Van der Tak, D., & Biney, A. (2020). Exploring Conscientious Objection to Abortion Among Health Providers in Ghana. International perspectives on sexual and reproductive health, 46, 51–59. https://doi.org/10.1363/46e8920; Keogh, L. A., Gillam, L., Bismark, M., McNamee, K., Webster, A., Bayly, C., & Newton, D. (2019). Conscientious objection to abortion, the law and its implementation in Victoria, Australia: perspectives of abortion service providers. BMC medical ethics, 20(1), 11. https://doi.org/10.1186/s12910-019-0346-1; Harries, J., Cooper, D., Strebel, A., & Colvin, C. J. (2014). Conscientious objection and its impact on abortion service provision in South Africa: a qualitative study. Reproductive health, 11(1), 16. https://doi.org/10.1186/1742-4755-11-16

[12] These effects are investigated in the legal evaluation studies referenced in footnotes 11 and 12.

**Causal Process**	**Intermediary or Primary Outcome(s)**	**Selection of Relevant Studies**
delay in obtaining lawful abortion<br>(*Pathway B*)		
Pregnant individual presents to a provider unwilling to perform abortion<br>(*Pathway C*)	Pregnant individual locates a willing provider on their own or through referral	*See Model IX for Delay*<br>*See Model X for Cost*
	Pregnant individual does not find a willing provider via referral or otherwise	*See Table XI for Unintended Childbirth*<br>*See Table XII for Legally Prohibited Abortion*

IX. Delay

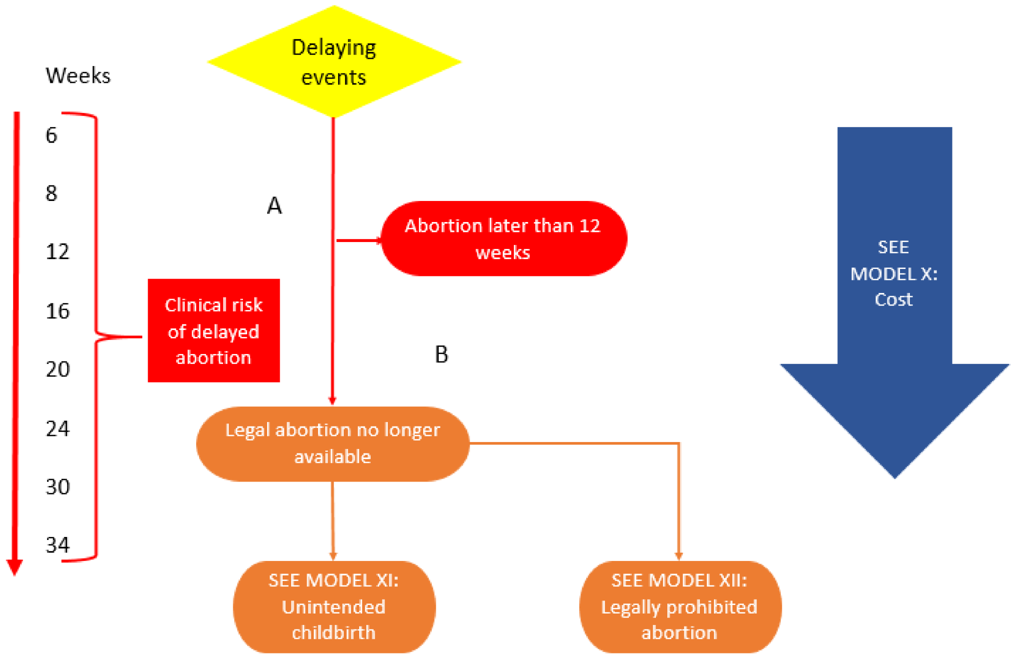

Figure IX. Delay: Causal Logic Model

This model depicts pathways related to delay in obtaining abortion care. Law can create delay on its own or in combination with other factors such as time to identify the pregnancy, financial barriers, and travel to a clinic. Delaying abortion care can lead to negative health outcomes for the pregnant individual, as well as increased costs associated with abortion at a later gestational age.

Pathway A (*red*) depicts delay leading to changes in clinical options for abortion, and an increasing risk of clinical complications as gestational age increases.

Pathway B (*orange*) depicts delay that leads to inability to obtain an abortion. This can result in the unintended birth of a child, or an abortion outside of legal parameters, including a self-managed abortion.

Each of these pathways may also contribute to increases in costs.

Table IX reports research we identified through the IDEAL process that provides findings on these processes and/or outcomes.

Table IX. Delay: Examples of Research on Identified Causal Pathways

Burris S, *et al. BMJ Global Health* 2021; 6:e005120. doi: 10.1136/bmjgh-2021-005120

Causal Process	Intermediary or Primary Outcome(s)	Selection of Relevant Studies
Delay in the procedures reduces clinical options for abortion as it pushes the abortion to later stages of gestation (*Pathway A*)	Increasing risk of clinical complications with increasing gestational age	• Zane, S., et al., Abortion-Related Mortality in the United States: 1998–2010. *Obstetrics & Gynecology*, 2015. 126(2).<br>• Bartlett, L.A., et al., Risk factors for legal induced abortion-related mortality in the United States. *Obstet Gynecol*, 2004. 103(4): p. 729-37.
Delay precludes abortion access (*Pathway B*)	Abortion unavailable due to legal, logistical or financial barriers	• Upadhyay, U. D., Weitz, T. A., Jones, R. K., Barar, R. E., & Foster, D. G. (2014). Denial of abortion because of provider gestational age limits in the United States. *American Journal of Public Health*, 104(9), 1687–1694. https://doi.org/10.2105/AJPH.2013.301378<br>• Janiak, E., Kawachi, I., Goldberg, A., & Gottlieb, B. (2014). Abortion barriers and perceptions of gestational age among women seeking abortion care in the latter half of the second trimester. *Contraception*, 89(4), 322–327. https://doi.org/10.1016/j.contraception.2013.11.009<br>• Drey, E. A., Foster, D. G., Jackson, R. A., Lee, S. J., Cardenas, L. H., & Darney, P. D. (2006). Risk factors associated with presenting for abortion in the second trimester. *Obstetrics and gynecology*, 107(1), 128–135. https://doi.org/10.1097/01.AOG.0000189095.32382.d0<br>• Barr-Walker, J., Jayaweera, R. T., Ramirez, A. M., & Gerdts, C. (2019). Experiences of women who travel for abortion: A mixed methods systematic review. PloS one, 14(4), e0209991. https://doi.org/10.1371/journal.pone.0209991

X. Cost

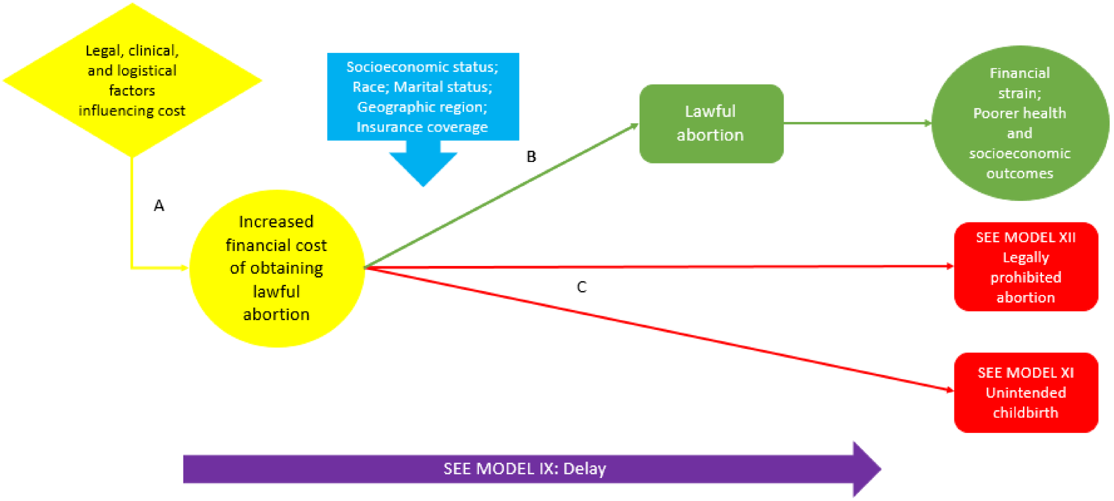

Figure X. Cost: Causal Logic Model

This model depicts pathways related to increased financial costs of obtaining abortion. Cost of an abortion can be a significant barrier to obtaining care and can exacerbate negative health and socioeconomic outcomes for the pregnant individual and their family even after a safe abortion. Where lawful abortion is unavailable, costs associated with legally prohibited abortion or unintended childbirth can be even more burdensome.

Pathway A (*yellow*) depicts the impact of legal, clinical, and logistical factors depicted in other models on the costs associated with obtaining abortion. The impact of cost is mediated by demographic factors such as socioeconomic status, marital status, and geographic location, as well as insurance coverage. As shown in Pathway B, increased financial cost may not preclude obtaining a lawful abortion, but may entail financial strain for the individual. Financial hardship can be serious, and long-term, and may include forgoing the payment of critical bills and utilities or borrowing money from family and friends in order to afford an abortion. Due to the cyclical nature of abortion costs, financial hardship can lead to more costs, such as interest on loans, ultimately leading to poorer health.

Pathway C (*red*) depicts inability to obtain an abortion because of cost leading to unintended childbirth or an abortion outside legal parameters. Unintended pregnancy and childbirth can lead to more costs through providing necessities for raising a child as well as costs associated with carrying the pregnancy to term, including complications during childbirth such as low birth weight, premature birth, and/or maternal morbidity and mortality.

Surmounting the barriers imposed by higher costs may cause delay in obtaining an abortion.

Table X reports research we identified through the IDEAL process that provides findings on these processes and/or outcomes.

Table X. Cost: Examples of Research on Identified Causal Pathways

Causal Process	Intermediary or Primary Outcome(s)	Selection of Relevant Studies
Legal requirements or processes, clinical and logistical factors influence costs associated with obtaining abortion (*Pathway A*)	Higher abortion-related costs	• Jerman, J., & Jones, R. K. (2014). Secondary Measures of Access to Abortion Services in the United States, 2011 and 2012: Gestational Age Limits, Cost, and Harassment. *Womens Health Issues*, 24(4). doi: 10.1016/j.whi.2014.05.002<br>• Jones, R. K., Upadhyay, U. D., & Weitz, T. A. (2013). At what cost? Payment for abortion care by U.S. women. Women's health issues : official publication of the Jacobs Institute of Women's Health, 23(3), e173–e178. https://doi.org/10.1016/j.whi.2013.03.001<br>• Shankar, M., Black, K. I., Goldstone, P., Hussainy, S., Mazza, D., Petersen, K., Lucke, J., & Taft, A. (2017). Access, equity and costs of induced abortion services in Australia: a cross-sectional study. Australian and New Zealand *journal of public health*, 41(3), 309–314. https://doi.org/10.1111/1753-6405.12641<br>• Huynh, L., McCoy, M., Law, A., Tran, K. N., Knuth, S., Lefebvre, P., Sullivan, S., & Duh, M. S. (2013). Systematic literature review of the costs of pregnancy in the US. PharmacoEconomics, 31(11), 1005–1030. https://doi.org/10.1007/s40273-013-0096-8
Increased abortion-related costs interact with individual socio-economic conditions and other contextual factors to influence longer-term well-being after lawful abortion is obtained (*Pathway B*)	Serious and long-term financial hardship	• Lince-Deroche, N., Constant, D., Harries, J., Blanchard, K., Sinanovic, E., & Grossman, D. (2015). *The costs of accessing abortion in South Africa: women's costs associated with second-trimester abortion services in Western Cape Province*. Contraception, 92(4), 339–344. doi: 10.1016/j.contraception.2015.06.029<br>• Foster, D. G., Biggs, M. A., Ralph, L., Gerdts, C., Roberts, S., & Glymour, M. M. (2018). Socioeconomic Outcomes of Women Who Receive and Women Who Are Denied Wanted Abortions in the United States. *American journal of public health*, 108(3), 407–413. https://doi.org/10.2105/AJPH.2017.304247<br>• Raidoo, S., Tschann, M., Kaneshiro, B., & Sentell, T. (2020). Impact of Insurance Coverage for Abortion in Hawai'i on Gestational Age at

Causal Process	Intermediary or Primary Outcome(s)	Selection of Relevant Studies
		Presentation and Type of Abortion, 2010-2013. *Hawai'i journal of health & social welfare*, 79(4), 117–122.
Increased abortion-related costs interact with individual socio-economic conditions and other contextual factors to prevent access to a lawful abortion (Pathway C)	Unintended childbirth or legally prohibited abortion	• Jones, R. K., & Kavanaugh, M. L. (2011). Changes in Abortion Rates Between 2000 and 2008 and Lifetime Incidence of Abortion. *Obstetrics & Gynecology*,117(6), 1358–1366. doi: 10.1097/aog.0b013e31821c405e<br>• Roberts, S. C., Gould, H., Kimport, K., Weitz, T. A., & Foster, D. G. (2014). Out-of-pocket costs and insurance coverage for abortion in the United States. *Women's health issues : official publication of the Jacobs Institute of Women's Health*, 24(2), e211–e218. https://doi.org/10.1016/j.whi.2014.01.003<br>*See Table XI for Unintended Childbirth*<br>*See Table XII for Legally Prohibited Abortion*

XI. Unintended Childbirth

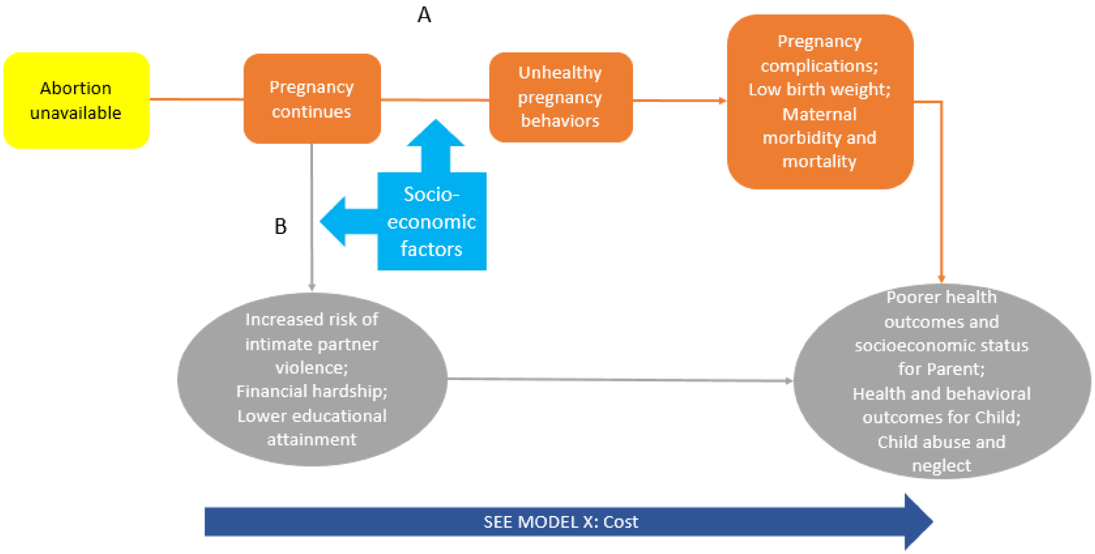

Figure XI. Unintended Childbirth: Causal Logic Model

This model depicts pathways and outcomes related to unintended pregnancy and childbirth as the result of being denied or otherwise being unable to access abortion. Unintended pregnancy and childbirth may be associated with negative health and socioeconomic impacts for the pregnant individual as well as their families and existing children.

Pathway A (*orange*): This pathway depicts the effects of socioeconomic stressors, including lack of health care access and economic strain, on the ability of the pregnant individual to adopt healthy pregnancy behaviors (such as abstaining from smoking) or get timely pre-natal care. These in turn affect the health of the pregnancy and the child, and may contribute to longer-term poorer outcomes for the pregnant individual and the child.

Pathway B (*gray*): This pathway depicts the increased risks of interpersonal, financial and educational problems for the pregnant individual carrying an undesired pregnancy to term. Like health effects, these are mediated by socio-economic status and can lead to long term poorer health and social outcomes.

Table XI reports research we identified through the IDEAL process that provides findings on these processes and/or outcomes.

Table XI. Unintended Childbirth: Examples of Research on Identified Causal Pathways

Burris S, *et al. BMJ Global Health* 2021; 6:e005120. doi: 10.1136/bmjgh-2021-005120

Causal Process	Intermediary or Primary Outcome(s)	Selection of Relevant Studies
Unintended pregnancy and socioeconomic stressors interact to increase the risk of unhealthy behavior and lack of resources during pregnancy (*Pathway A*)	Unhealthy pregnancy behaviors, resource limitations, pregnancy complications and poorer birth outcomes	• Scholl, T.O., M.L. Hediger, and D.H. Belsky, *Prenatal care and maternal health during adolescent pregnancy: a review and meta-analysis*. J Adolesc Health, 1994. 15(6): p. 444-56.<br>• Gerdts, C., et al., *Side Effects, Physical Health Consequences, and Mortality Associated with Abortion and Birth after an Unwanted Pregnancy*. Women's Health Issues, 2016. 26(1): p. 55-59.
Unintended pregnancy and socioeconomic stressors interact to increase the risk of interpersonal, financial and educational problems for the pregnant individual (*Pathway B*)	Intimate partner violence, financial stress, and lower educational attainment	• Miller, S., L.R. Wherry, and D.G. Foster, *What Happens after an Abortion Denial? A Review of Results from the Turnaway Study*. AEA Papers and Proceedings, 2020. 110: p. 226-30.<br>• Miller, S., L.R. Wherry, and D.G. Foster, *The Economic Consequences of Being Denied an Abortion*. National Bureau of Economic Research Working Paper Series, 2020. 26662.
Unintended pregnancy interacting with socioeconomic factors leads to long-term poorer health and social outcomes for pregnant individual and the child (*Pathways A & B*)	Socioeconomic factors may mediate negative outcomes related to unintended pregnancy and childbirth	• Foster, D.G., et al., *Effects of Carrying an Unwanted Pregnancy to Term on Women's Existing Children*. J Pediatr, 2019. 205: p. 183-189.e1<br>• Foster, D.G., et al., *Comparison of Health, Development, Maternal Bonding, and Poverty Among Children Born After Denial of Abortion vs After Pregnancies Subsequent to an Abortion*. JAMA Pediatr, 2018. 172(11): p. 1053-1060.<br>• Foster, D.G., et al., *Socioeconomic Outcomes of Women Who Receive and Women Who Are Denied Wanted Abortions in the United States*. American Journal of Public Health, 2018. 108(3): p. 407-413.<br>• Bahk, J., Yun, S. C., Kim, Y. M., & Khang, Y. H. (2015). Impact of unintended pregnancy on maternal mental health: a causal analysis using follow up data of the Panel Study on Korean Children (PSKC). *BMC pregnancy and childbirth*, 15, 85. https://doi.org/10.1186/s12884-015-0505-4

Causal Process	Intermediary or Primary Outcome(s)	Selection of Relevant Studies
		• Finer, L. B., & Zolna, M. R. (2011). Unintended pregnancy in the United States: incidence and disparities, 2006. Contraception, 84(5), 478–485. https://doi.org/10.1016/j.contraception.2011.07.013<br>• Foster, D.G., et al., *Effects of Carrying an Unwanted Pregnancy to Term on Women's Existing Children*. J Pediatr, 2019. 205: p. 183-189.e1

XII. Legally Prohibited Abortion

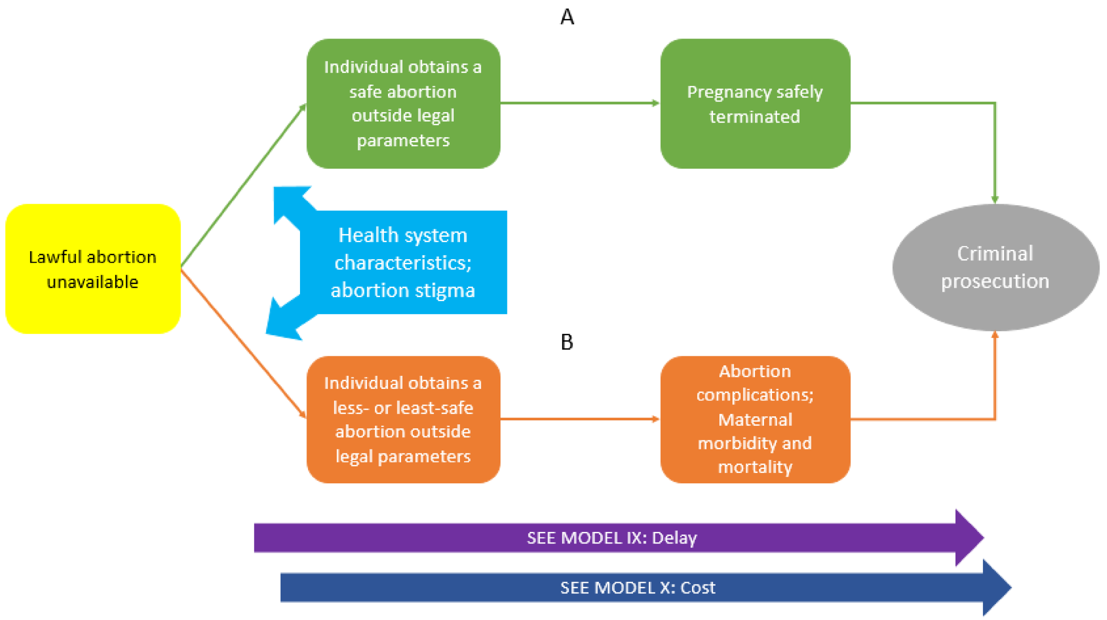

Figure XII. Legally Prohibited Abortion: Causal Logic Model

People who are unable to obtain a safe, legal abortion may resort to seeking an abortion outside legal parameters. The legal prohibition of abortion does not necessarily mean that such an abortion will be unsafe. The WHO defines a safe abortion as one that uses a recommended method appropriate to the pregnancy duration, and that is provided or supported by a person who has the been trained in the necessary skills. An abortion is "less safe" when it only meets one of these criteria, and "least-safe" when it meets neither.[13] (This model does not show that a pregnant individual may also be forced by criminalization to carry an unintended pregnancy to term. See Figure XI.)

Pathway A *(green)*: This pathway describes a pregnant individual who does not qualify for a legal abortion but obtains a safe abortion outside of legal requirements. This option depends on features of the abortion service-delivery environment, including the availability and accessibility of medication for self-managed abortion, and of properly trained providers willing to perform abortions using a recommended method in a safe setting. A self-managed abortion by a person who has the necessary information, properly using the combination of mifepristone and misoprostol, is considered to be a safe abortion. A safe termination outside of legal parameters may result in criminal prosecution.

[13] See Sedgh, G., Filippi, V., Owolabi, O. O., Singh, S. D., Askew, I., Bankole, A., . . . MacDonagh, S. (2016). Insights from an expert group meeting on the definition and measurement of unsafe abortion. International Journal of Gynecology & Obstetrics, 134(1), 104-106. doi: https://doi.org/10.1016/j.ijgo.2015.11.017. See generally Moseson, H., Herold, S., Filippa, S., Barr-Walker, J., Baum, S. E., & Gerdts, C. (2020). Self-managed abortion: A systematic scoping review. Best Practice & Research Clinical Obstetrics & Gynaecology, 63, 87-110. doi: https://doi.org/10.1016/j.bpobgyn.2019.08.002

Pathway B *(orange)*: This pathway describes a pregnant individual who does not qualify for a legal abortion but obtains an abortion less safe or least safe. Fear of abortion stigma may influence an individual's decision to obtain unsafe abortion and deter them from seeking care for complications. Lack of health services and infrastructure may also factor into unsafe abortions. Abortion complications and maternal morbidity or mortality that result from less-safe or least- safe abortions can lead to poorer health and socioeconomic outcomes, as well as increased costs. A less or least safe may also result I criminal prosecution. [14]

Delayed care and increased results may arise in both pathways.

Table XII reports research we identified through the IDEAL process that provides findings on these processes and/or outcomes.

Table XII. Legally Prohibited Abortion: Examples of Research on Identified Causal Pathways

Causal Process	Intermediary or Primary Outcome(s)	Selection of Relevant Studies
Health system characteristics including the availability of abortion information and abortion stigma interact with law prohibiting abortion to afford access to a safe abortion outside legal parameters (*Pathway A*)	Pregnancy is terminated through a safe abortion	• Ganatra et al. *Global, regional, and subregional classification of abortions by safety, 2010–14: estimates from a Bayesian hierarchical model*. The Lancet, Vol. 390, Issue 10110, Nov. 25, 2017.<br>• Ngo, T.D., et al., *Comparative effectiveness, safety and acceptability of medical abortion at home and in a clinic: a systematic review*. Bull World Health Organ, 2011. 89(5): p. 360-70.<br>• Gambir, K., et al., Self-administered versus provider-administered medical abortion. Cochrane Database Syst Rev, 2020. 3: p. CD013181.<br>• Shellenberg, K.M., et al., Social stigma and disclosure about induced abortion: results from an exploratory study. Global Public Health, 2011. 6(Suppl. 1): p. 111-125.<br>• Fernandez, M.M., et al., Assessing the global availability of misoprostol. International Journal of Gynecology & Obstetrics, 2009. 105(2): p. 180-186<br>• Ngo, T.D., et al., Comparative effectiveness, safety and acceptability of medical abortion at home and in a clinic: a systematic review. Bull World Health Organ, 2011. 89(5): p. 360-70.<br>• Rodriguez, K., & Strickler, J. (1999). Clandestine abortion in Latin America: provider

[14] For a comprehensive census and analysis of criminal abortion cases in the United States, see Paltrow, L. M., & Flavin, J. (2013). Arrests of and Forced Interventions on Pregnant Women in the United States, 1973-2005: Implications for Women's Legal Status and Public Health. Journal of Health Politics, Policy & Law, 38(2), 299–343.

Causal Process	Intermediary or Primary Outcome(s)	Selection of Relevant Studies
		perspectives. Women & health, 28(3), 59–76. https://doi.org/10.1300/J013v28n03_05
Health system characteristics including the availability of abortion information and abortion stigma interact with law prohibiting abortion to afford access to a less-safe or leas- safe abortion outside legal parameters (*Pathway B*)	Pregnancy is terminated through a less-safe or least-safe abortion	• Shamsi, S., Mirza, T. T., Shejuti, T. R., Nigar, K., Nahar, S., Begum, S., Sharmin, T., Panna, L. K., Islam, N., & Jahan, T. (2020). An Overview of Unsafe Abortion: Patterns and Outcomes in a Tertiary Level Hospital. *Mymensingh medical journal* : MMJ, 29(3), 523–529.<br>• Srinil S. (2011). Factors associated with severe complications in unsafe abortion. *Journal of the Medical Association of Thailand = Chotmaihet thangphaet*, 94(4), 408–414.<br>• Koblinsky M, Chowdhury ME, Moran A, Ronsmans C. Maternal morbidity and disability and their consequences: neglected agenda in maternal health. J *Health Popul Nutr*. 2012;30(2):124–130<br>• Yegon, E.K., et al., Understanding abortion-related stigma and incidence of unsafe abortion: experiences from community members in Machakos and Trans Nzoia counties Kenya. The Pan African medical journal, 2016. 24: p. 258-258.<br>• Parmar, D., Leone, T., Coast, E., Murray, S. F., Hukin, E., & Vwalika, B. (2017). Cost of abortions in Zambia: A comparison of safe abortion and post abortion care. *Global public health*, 12(2), 236–249. https://doi.org/10.1080/17441692.2015.1123747<br>• Levin, C., Grossman, D., Berdichevsky, K., Diaz, C., Aracena, B., Garcia, S. G., & Goodyear, L. (2009). Exploring the costs and economic consequences of unsafe abortion in Mexico City before legalisation. *Reproductive health matters*, 17(33), 120–132. https://doi.org/10.1016/S0968-8080(09)33432-1<br>• Zafar, H., Ameer, H., Fiaz, R., Aleem, S., & Abid, S. (2018). Low Socioeconomic Status Leading to Unsafe Abortion-related Complications: A Third-world Country Dilemma. *Cureus,* 10(10), e3458. https://doi.org/10.7759/cureus.3458

Causal Process	Intermediary or Primary Outcome(s)	Selection of Relevant Studies
Abortion outside of legal parameters increases legal risks of abortion (*Pathways A & B*)	Criminal prosecution	• Casas Becerra, Lidia. 1997. "Women Prosecuted and Imprisoned for Abortion in Chile." *Reproductive Health Matters* 5 (9): 29–36.10.1016/S0968-8080(97)90003-3.<br>• Melese, T., Habte, D., Tsima, B. M., Mogobe, K. D., Chabaesele, K., Rankgoane, G., Keakabetse, T. R., Masweu, M., Mokotedi, M., Motana, M., & Moreri-Ntshabele, B. (2017). High Levels of Post-Abortion Complication in a Setting Where Abortion Service Is Not Legalized. *PloS one*, 12(1), e0166287. https://doi.org/10.1371/journal.pone.0166287<br>• McNaughton HL, Mitchell EM, Hernandez EG, Padilla K, Blandon MM. Patient privacy and conflicting legal and ethical obligations in El Salvador: reporting of unlawful abortions. *Am J Public Health*. 2006;96(11):1927–1933<br>• Paine J, Noriega RT, Puga AL. Using litigation to defend women prosecuted for abortion in Mexico: challenging state laws and the implications of recent court judgments. *Reprod Health Matters*. 2014 Nov;22(44):61-9. doi: 10.1016/S0968-8080(14)44800-6. PMID: 25555763.<br>• Umuhoza C, Oosters B, van Reeuwijk M, Vanwesenbeeck I. Advocating for safe abortion in Rwanda: how young people and the personal stories of young women in prison brought about change. *Reprod Health Matters*. 2013 May;21(41):49-56. doi: 10.1016/S0968-8080(13)41690-7. PMID: 23684187.
